# Evaluation of a COVID-19 convalescent plasma program at a U.S. academic medical center

**DOI:** 10.1371/journal.pone.0277707

**Published:** 2022-12-08

**Authors:** Heather B. Root, Matt Gilleskie, Chih-Huan Lu, Andrew Gilmore, Mariama Evans, Bridget G. Nelson, William Johnson, Brian Gurney, JoAnn Kuruc, Alena J. Markmann, Amir H. Barzin, David A. Wohl, William A. Fischer, Yara A. Park, Susan Weiss, Sonia Napravnik, Ralph Baric, Aravinda M. de Silva, Anne M. Lachiewicz, David van Duin, David M. Margolis, Michael E. Herce, Luther A. Bartelt

**Affiliations:** 1 Division of Infectious Diseases, Department of Medicine, The University of North Carolina School of Medicine, Chapel Hill, NC, United States of America; 2 Division of Infectious Diseases, Department of Medicine, Albert Einstein College of Medicine, Bronx, NY, United States of America; 3 Department of Medicine, The University of North Carolina, Chapel Hill, NC, United States of America; 4 Department of Pathology and Laboratory Medicine, The University of North Carolina School of Medicine, Chapel Hill, NC, United States of America; 5 UNC HIV Cure Center, The University of North Carolina School of Medicine, Chapel Hill, NC, United States of America; 6 Department of Family Medicine, The University of North Carolina School of Medicine, Chapel Hill, NC, United States of America; 7 Division of Pulmonary and Critical Care Medicine, The University of North Carolina, Chapel Hill, NC, United States of America; 8 Department of Pathology, Atrium Health Carolinas Medical Center, Charlotte, NC, United States of America; 9 Department of Epidemiology, The University of North Carolina, Chapel Hill, NC, United States of America; 10 Department of Microbiology and Immunology, The University of North Carolina School of Medicine, Chapel Hill, NC, United States of America; University of KwaZulu-Natal, SOUTH AFRICA

## Abstract

Amidst the therapeutic void at the onset of the COVID-19 pandemic, a critical mass of scientific and clinical interest coalesced around COVID-19 convalescent plasma (CCP). To date, the CCP literature has focused largely on safety and efficacy outcomes, but little on implementation outcomes or experience. Expert opinion suggests that if CCP has a role in COVID-19 treatment, it is early in the disease course, and it must deliver a sufficiently high titer of neutralizing antibodies (nAb). Missing in the literature are comprehensive evaluations of how local CCP programs were implemented as part of pandemic preparedness and response, including considerations of the core components and personnel required to meet demand with adequately qualified CCP in a timely and sustained manner. To address this gap, we conducted an evaluation of a local CCP program at a large U.S. academic medical center, the University of North Carolina Medical Center (UNCMC), and patterned our evaluation around the dimensions of the Reach, Effectiveness, Adoption, Implementation, and Maintenance (RE-AIM) framework to systematically describe key implementation-relevant metrics. We aligned our evaluation with program goals of reaching the target population with severe or critical COVID-19, integrating into the structure of the hospital-wide pandemic response, adapting to shifting landscapes, and sustaining the program over time during a compassionate use expanded access program (EAP) era and a randomized controlled trial (RCT) era. During the EAP era, the UNCMC CCP program was associated with faster CCP infusion after admission compared with contemporaneous affiliate hospitals without a local program: median 29.6 hours (interquartile range, IQR: 21.2–48.1) for the UNCMC CCP program versus 47.6 hours (IQR 32.6–71.6) for affiliate hospitals; (P<0.0001). Sixty-eight of 87 CCP recipients in the EAP (78.2%) received CCP containing the FDA recommended minimum nAb titer of ≥1:160. CCP delivery to hospitalized patients operated with equal efficiency regardless of receiving treatment via a RCT or a compassionate-use mechanism. It was found that in a highly resourced academic medical center, rapid implementation of a local CCP collection, treatment, and clinical trial program could be achieved through re-deployment of highly trained laboratory and clinical personnel. These data provide important pragmatic considerations critical for health systems considering the use of CCP as part of an integrated pandemic response.

## Introduction

As SARS-CoV-2 infections rapidly swept the globe in early 2020 [1, 2], hospitals were faced with caring for an influx of severely ill patients without a therapeutic standard of care. Although antiviral and immunomodulatory agents were thought to be possible therapeutic options, there were initially few evidence-based treatments available. Further, the supply of novel anti-SARS-CoV-2 anti-virals and patient access to enroll in randomized controlled trials (RCT) was limited. COVID-19 convalescent plasma (CCP) emerged as a leading therapeutic option. CCP was considered scalable to meet the rapidly increasing demands across both large and smaller medical centers. However, like other early therapeutics, the literature at the time suggested conflicting evidence of its potential efficacy [3].

Enthusiasm for the therapeutic benefits of CCP included experimental models of sarbecovirus pathogenesis and preliminary reports suggesting antibodies from recovered individuals neutralize SARS-CoV-2, interrupting viral replication and virus-mediated damage in patients with active infection [4, 5]. Additionally, convalescent plasma had been safely used in other respiratory viral infections including SARS-CoV-1, MERS-CoV, and 2009 H1N1 Influenza [6–9]. CCP collection and distribution could also leverage a broad network of blood banking capabilities where available, thereby making the therapeutic use of CCP immediately scalable in well-resourced settings.

In the United States (U.S.), an Expanded Access Program (EAP) for CCP treated more than 100,000 participants hospitalized with severe COVID-19 [10–12]. Large regional and national blood collection centers reported rapid implementation and mobilization efforts to recruit, collect, and distribute CCP in diverse healthcare settings, demonstrating that large blood donation networks can deliver CCP at scale. The University of North Carolina Medical Center (UNCMC), and other academic medical centers capable of collecting plasma, also developed local programs for on-site use [13]. These local programs were designed to maximize institutional resources for community benefit, align with translational and clinical research, and reduce the demand on national blood donation banks. Additionally, there may also be clinical benefits associated with using locally-sourced plasma that may better represent regionally-specific, prevalent SARS-CoV-2 variants [14].

Although implementation is typically focused on therapies with proven clinical efficacy, in the context of this novel rapidly spreading and fatal virus, therapeutic programs, like the UNCMC CCP program, had to implement potential life-saving therapeutics before clinical effectiveness was known. Thus, in this report, we evaluate the impact of the UNCMC CCP program, patterning our evaluation after the dimensions of the Reach, Effectiveness, Adoption, Implementation, and Maintenance (RE-AIM) framework, which has been used extensively to guide program evaluations in clinical settings, including unconventional clinical settings [15–19]. We document the core components and personnel needed in health systems deploying CCP in response to COVID-19. We describe the UNCMC CCP program’s performance against pre-specified program goals of reaching a target population with severe or critical COVID-19, integrating into the structure of the hospital-wide pandemic response and individual care teams, adapting to shifting landscapes, and moving toward sustainability over time. Key implementation-relevant metrics and process measures are compared between CCP delivery for emergency compassionate use versus participation in a RCT [20, 21].

## Methods

### Ethics statement

CCP was collected and stored at UNCMC in accordance with U.S. Food and Drug Administration (FDA) guidelines [22] and the UNCMC Blood Donation Center (BDC) standard operating procedures. Other aspects of CCP studies were performed under the oversight of the UNC Institutional Review Board (IRB), in accordance with the FDA EAP for CCP, and in accordance with UNC’s CCP RCT protocol and UNC IRB 20–1544 (https://clinicaltrials.gov/ct2/show/NCT04524507). Review of electronic health record data for patients with COVID-19 at UNCMC and UNC-affiliated hospitals was done under a UNC IRB approved research study registry, UNC COVID Cohort (UNC IRB 20–1095). All patients, or their legally authorized representatives (LARs), gave informed consent, which in some cases occurred via a two-MD consent (described below).

### Study design, program evaluation, definitions, and metrics

Given prevailing expert opinion on CCP in early 2020, the primary *a priori* program goals for the UNC CCP program were to treat as many patients as were eligible and desired treatment, and treat patients within 72 hours of admission (as a proxy measure for program effectiveness). Because we had access to laboratory capabilities to perform neutralizing antibody (nAb) titers, we aimed to provide CCP consistent with the initial FDA-recommended guidance of nAb titer >1:160 or > 1:80 if supply was limited, as often as possible [22]. As expert opinion evolved during the pandemic, we added a goal of treating within 10 days of symptoms onset (a more biologically and clinically relevant time-bound measure). These goals extended throughout the EAP era of CCP administration (April 2020—August 2020). When the FDA transitioned from an EAP to an emergency use authorization (EUA) mechanism in late August 2020, we integrated CCP delivery into clinical care via an investigator-initiated RCT (rather than the EUA). Our RCT goal was to enroll without delaying COVID-19 treatment access for study participants (i.e. to deliver CCP as quickly via the RCT as we could deliver CCP via other compassionate use mechanisms). The RCT was intentionally designed to provide CCP with nAb titer ≥ 1:160 and within 10 days of symptoms onset (https://clinicaltrials.gov/ct2/show/NCT04524507). Additionally, we had the goals of leveraging blood banking and regulatory resources to facilitate affiliate hospital uptake of CCP as well as avoiding unintended negative effects from the CCP program on receiving COVID-19 treatment, which we assessed via using time to remdesivir as a comparator. We generally organized our evaluation of the UNCMC CCP program against these goals according to definitions patterned after the evaluative dimensions of the RE-AIM framework [15], with specific metrics for our program detailed in Table 1.

**Table 1:** Reach, Effectiveness, Adoption, Implementation and Maintenance (RE-AIM) constructs.

Dimension	Definition	Primary metrics
**Reach**	• The ability of the CCP program to reach the target population. • The extent to which those reached are representative of those most at risk.	• Proportion of patients consenting for CCP who received CCP during the EAP era. • Representativeness of admitted patients who received CCP versus those who did not receive CCP during the EAP era.
**Effectiveness**	• The impact of the CCP program on time from admission to CCP infusion at UNCMC versus affiliate hospitals. • Integration of the program into routine clinical operations without disruption of COVID-19 care.	• The proportion of patients at UNCMC who received CCP within 10 days of symptom onset in the EAP era. • Proportion of patients who received CCP infusion within 72 hours of admission at UNCMC versus affiliate hospitals in the EAP era. • Time to remdesivir in CCP recipients and non-recipients at UNCMC in the EAP era.
**Adoption**	• The uptake of CCP by hospitals within the UNC Health System. • At UNCMC, differential uptake of CCP by location (ICU vs non-ICU wards), and efficiency of CCP administration by location, time, and day of admission.	• Affiliate hospital participation in the EAP and EUA. • Number of ICU and non-ICU patients receiving CCP at UNCMC during the EAP era.
**Implementation**	• Staffing and resources needed to collect and infuse plasma via the UNCMC CCP program and the consistency of the UNCMC CCP program to deliver CCP per FDA-recommended minimum neutralizing antibody titer.	• Number of core personnel required • Proportions of transfusable units collected and whether units were qualified as meeting FDA-recommended minimum neutralizing antibody titer (1:160). • Assess consistency of program delivery over time as program adapted to emerging guidelines and data (directing toward earlier treatment in less severe patients).
**Maintenance**	• Institutionalization of the program at UNCMC.	• Proportion of patients receiving CCP as a function of month of admission. • Cumulative units collected and transfused over time and cumulative patients transfused over time during the EAP, including units sourced from either UNCMC or national supplier.

CCP = COVID-19 Convalescent Plasma, UNCMC = University of North Carolina Medical Center, EAP = Expanded Access Program, EUA = Emergency Use Authorization, ICU = Intensive Care Unit, FDA = Food and Drug Administration

### Study site and program characteristics

UNCMC is a quaternary care U.S. academic medical center with 950 hospital beds (43 medical intensive care unit (ICU) beds) [23, 24] located in Chapel Hill, North Carolina. UNCMC is one of 11 hospitals in the UNC Health system encompassing a total of >3400 beds, including 403 ICU beds. Simultaneous with the FDA EAP, UNCMC developed a local CCP program. The primary goals of the program were to provide CCP to UNCMC COVID-19 patients, through an RCT for those eligible and interested in research participation, and through clinical care otherwise included via the compassionate use mechanisms (EAP and later the EUA). This program had three core components: 1) on-site donation, banking, and transfusion capacity, 2) on-site novel assays to directly measure the *functional* anti-viral activity of CCP, and 3) a dedicated clinical trials unit with key clinical research staff, study coordinators, and investigators. The timeline of the program is shown in S1 Fig.

#### CCP donor recruitment

Beginning March 16, 2020, plasma donors were recruited at UNCMC by direct laboratory test referrals as well as referrals from other local studies, media outlets, and social media, and from direct referrals [25]. Qualification for donation was in accordance with FDA guidelines [22] and required laboratory evidence of prior SARS-CoV-2 infection. All potential donors were contacted via phone for initial screening and if deemed eligible, scheduled for donation.

#### CCP collection and storage

At the UNCMC BDC, CCP was collected between April 11, 2020—February 24, 2021 (reported here through September 2, 2020). Plasma was collected by apheresis on dedicated donation days and stored on-site. Only patients admitted to UNCMC had access to the CCP collected at the UNCMC BDC. CCP was also available to U.S. hospitals through national and regional blood banks.

#### Mechanisms to receive CCP

**Fig 1:**
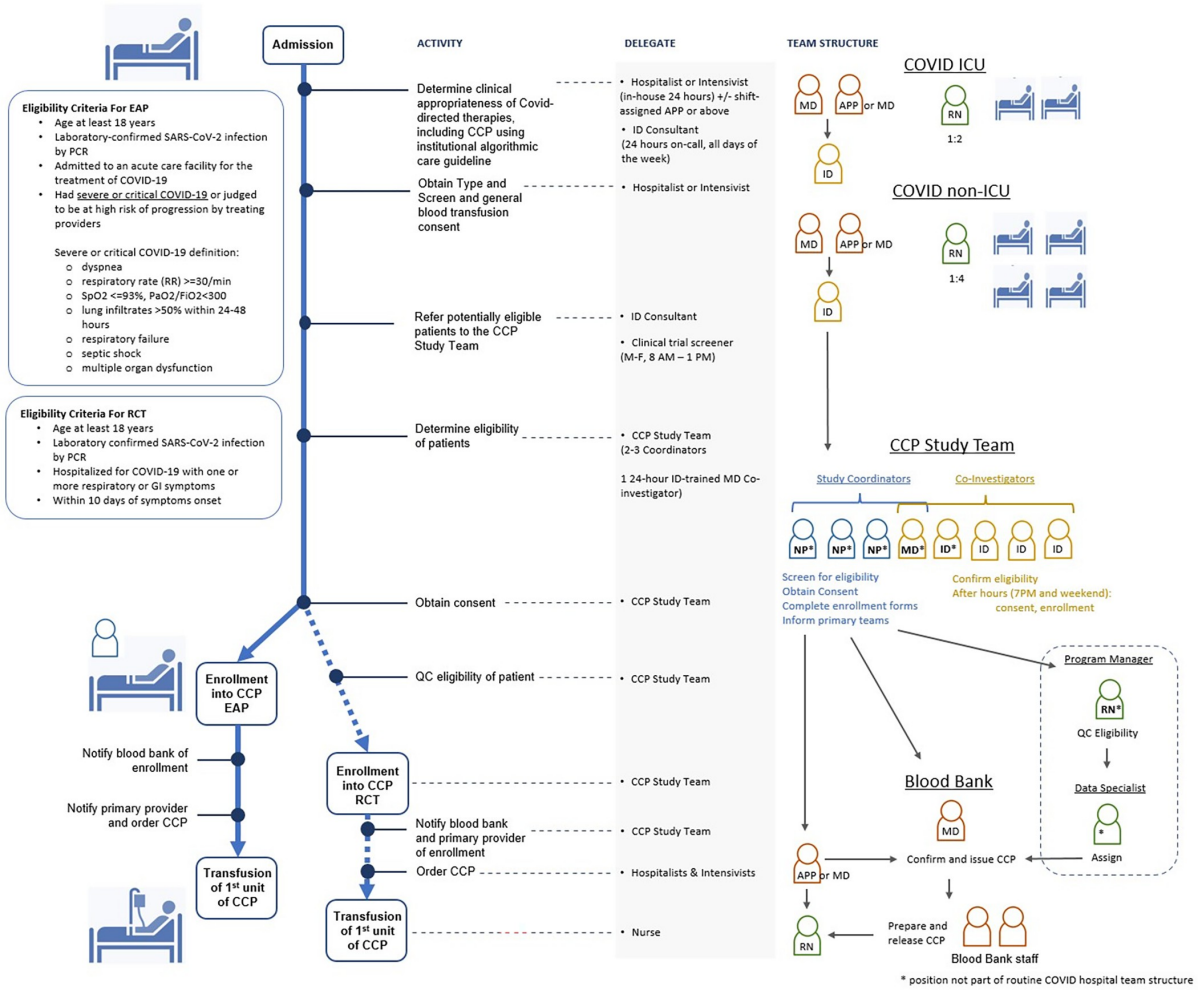
CCP administration process schematic. Steps, eligibility criteria, and personnel involved in administering COVID-19 Convalescent Plasma (CCP) in the inpatient setting at The University of North Carolina Medical Center (UNCMC). The major process events of admission, enrollment, and infusion depicted in Fig 1 are scaled proportionally to the median time intervals spent on these activities at UNCMC. EAP = expanded access program, PCR = polymerase chain reaction, RCT = randomized controlled trial, GI = gastrointestinal, ICU = intensive care unit, ID = infectious diseases, MD = medical doctor, APP = advanced practice provider, RN = registered nurse, NP = nurse practitioner, QC = quality control.

Initially, patients admitted to UNCMC received CCP via the FDA EAP. Later, an FDA EUA for CCP became available. CCP was also available via UNCMC’s investigator-initiated RCT comparing the safety and clinical outcomes of patients who received CCP with at least the FDA-recommended minimum nAb titer of 1:160 versus those who received CCP containing nAb titers >1:640. Eligibility criteria for EAP, RCT, and EUA were followed according to respective protocols [26, 27] (Fig 1). Informed consent for the EAP or RCT was obtained directly from the participant or a LAR by the dedicated CCP study team. Consents were available in both electronic and paper format in English and Spanish. The two-MD consent was only available for EAP participants under emergency circumstances if the patient or LAR could not provide required consent. The EUA process required presentation of an FDA Fact Sheet and verbal consent with the patient and/or LAR. UNCMC participated in the CCP EAP from April 24, 2020 to August 29, 2020. The RCT operated between August 22, 2020 to December 4, 2020 [22, 26, 27].

#### Assessment and enrollment processes

Clinical evaluation of the patient was performed by the primary COVID-19 dedicated team of hospitalists, intensivists, and infectious diseases (ID) consultants following a regularly updated standardized hospital-wide treatment algorithm posted on the UNC intranet. The ID division instituted weekly COVID-19 treatment algorithm discussions to adapt clinical care practices to evolving literature and normative guidance. A dedicated study screening process was implemented in the hospital to provide equitable access to different treatment study protocols. The CCP study team integrated into this system providing admitting teams and ID consultants with the various eligibility criteria for receiving CCP (Fig 1). When appropriate candidates for CCP therapy were identified and approved by ID consultation, the CCP study team was contacted. The study team obtained consent and enrolled patients and then notified primary providers to proceed with CCP infusion.

#### Convalescent plasma viral neutralizing antibody titer assay

The anti-viral neutralization capability of CCP was performed by using remnant plasma collected during donation. The nAb titer of each unit was measured using a live reporter SARS-CoV-2-nLUC viral neutralization assay [28]. nAb testing was performed frequently in batches, every 1–2 weeks, to provide a sufficient supply of CCP with specified nAb titer.

### Study

#### Data collection

Data for UNCMC CCP recipients and non-recipients were collected from institutional electronic health records (EHR). A clinical cohort of all patients in the UNC Health system with suspected or confirmed COVID-19 was created, drawn from EHR data and medical record reviews. For demographics data, we included all patients ≥18 years of age, hospitalized during the EAP era between 4/11/2020 and 8/31/2020, with either laboratory confirmed SARS-CoV-2 infection (during hospitalization or ≤21 days of admission) or a COVID-19 diagnosis (during hospitalization). Data collected included patient demographics, comorbidities, COVID-19 therapeutics received, clinical course, and clinical outcomes. For infusion times among RCT enrollees at UNCMC (8/22/2020 through 12/4/2020) we manually extracted data from the EHR through the day of the last RCT enrollee in accordance UNC IRB 20–1095. Data on patients who received CCP through the EAP (4/11/2020 through 8/31/2020) and EUA (9/1/2020 through 12/4/2020) at UNC Health, but not at UNCMC (i.e., at affiliate hospitals), were manually collected from EHR records and included site, EAP or EUA participation, as well as dates and times of hospitalization and CCP transfusion.

#### Statistical analysis

Descriptive statistics were used to present study results, with median and interquartile range (IQR) used as measures of central tendency unless otherwise specified. We used Pearson’s Chi-squared, Mann-Whitney U and Kruskal-Wallis, one-way ANOVA with Tukey-Kramer post hoc tests to compare differences between groups, as appropriate, with two-sided P-values reported. P-values were adjusted for multiplicity. Analyses were conducted using SAS 9.4 (Cary, NC) and Graphpad Prism 9 (San Diego, CA).

## Results

### Reach

During the 18-week EAP participation period, 526 patients were admitted to the UNCMC with SARS-CoV-2 infection and 163 (30.9%) received CCP (Table 2). All patients who enrolled in the EAP received CCP. Most CCP recipients had severe or critical disease (N = 151; 93.8%) and 10 (6.2%) were considered at high-risk of progression to severe or critical disease. Compared to non-CCP recipients, CCP recipients were more likely to have more severe illness as measured by greater use of ICU-level supportive care. Hispanic ethnicity was associated with greater likelihood of receiving CCP (unadjusted for severity of COVID-19).

**Table 2:** CCP recipient and non-recipient characteristics.

Characteristic	Non-CCP recipients* (N = 363)	CCP EAP Recipients* (N = 163)	p-value
**Demographics**			
** Age (years), median (IQR)**	53.9 (38.5–66.4)	55.6 (46.1–65.5)	0.146
** 18–39, n (%)**	106 (29.2)	29 (17.8)	
** 40–64, n (%)**	159 (43.8)	91 (55.8)	
** 65–79, n (%)**	68 (18.7)	34 (20.9)	
** 80+, n (%)**	30 (8.3)	9 (5.5)	
** Sex, n (%)**			0.082
** Female**	199 (54.8)	76 (46.6)	
** Male**	164 (45.2)	87 (53.4)	
** Race, n (%)**			0.197
** White**	96 (26.4)	42 (25.8)	
** Black**	105 (28.9)	37 (22.7)	
** Other**	149 (41.0)	81 (49.7)	
** Unknown**	13 (3.6)	3 (1.8)	
** Ethnicity, n (%)**			0.020
** Hispanic**	138 (38.0)	84 (51.5)	
** Non-Hispanic**	212 (58.4)	78 (47.9)	
** Unknown**	13 (3.6)	1 (0.6)	
**Admission number per month, n (%)**			0.020
** April**	25 (6.9)	7 (4.3)	
** May**	69 (19.0)	30 (18.4)	
** June**	72 (19.8)	50 (30.7)	
** July**	99 (27.3)	48 (29.4)	
** August**	98 (27.0)	28 (17.2)	
**BMI, n (%)**			
** Median (IQR)**	29.2 (25.2–35.1)	32.2 (28.0–37.4)	<0.001
** <18.5**	7 (2.2)	4 (2.5)	
** 18.5 to <25**	71 (22.0)	12 (7.6)	
** 25 to <30**	95 (29.4)	44 (27.8)	
** 30 to <35**	68 (21.1)	41 (25.9)	
** 35 to <40**	38 (11.8)	31 (19.6)	
** 40 or higher**	44 (13.6)	26 (16.5)	
**Smoking Status, n (%)**			0.039
** Current (includes every day, some days, light, heavy)**	26 (7.3)	8 (5.1)	
** Former**	106 (29.6)	32 (20.4)	
** Never**	210 (58.7)	113 (72.0)	
** Unknown/never assessed**	16 (4.5)	4 (2.5)	
**Comorbidities, n (%)**			0.156
** Hypertension**	203 (55.9)	104 (63.8)	
** Diabetes**	147 (40.5)	92 (56.4)	
** CAD**	59 (16.3)	40 (24.5)	
** COPD**	41 (11.3)	12 (7.4)	
** Asthma**	31 (8.5)	22 (13.5)	
** Any comorbidity**	250 (68.9)	123 (75.5)	
**COVID-19 specific therapy, n (%)**			0.015
** Remdesivir**	70 (19.3)	124 (76.1)	<0.0001
** Dexamethasone**	72 (19.8)	92 (56.4)	<0.0001
** Tocilizumab**	3 (0.8)	3 (1.8)	
** Hydroxychloroquine**	8 (2.2)	3 (1.8)	
** Lopinavir/Ritonavir +/- Ribavirin**	17 (4.7)	10 (6.1)	
**COVID-19 specific therapy**			< 0.001
** Any COVID-19-specific therapy above**	122 (33.6)	142 (87.1)	
** None of the above**	241 (66.4)	21 (12.9)	
**Required Ventilation, n (%) **	58 (16.0)	54 (33.1)	< 0.001
**Required ECMO, n (%)**	13 (3.6)	15 (9.2)	0.008

*Categories do not sum to N due to missing data

IQR = interquartile range; n = number; chi-sq = chi squared test; BMI = body mass index; CAD–coronary artery disease; COPD = chronic obstructive pulmonary disorder; ECMO = extracorporeal membrane oxygenation

### Effectiveness

Among recipients with documented date of symptoms onset, 71.5% (N = 113/158) were treated within 10 days of symptom onset. Overall, 94.5% (N = 154/163) and 47.2% (N = 77/163) were treated within 10 and 3 days of diagnosis, respectively. Compared with CCP non-recipients, recipients were also more likely to receive at least one other COVID-19-directed therapy (87.1% vs 33.6%) including remdesivir (76.1% vs 19.3%, P<0.0001) and dexamethasone (56.4% vs 19.8%, P<0.0001) (Table 2).

**Fig 2:**
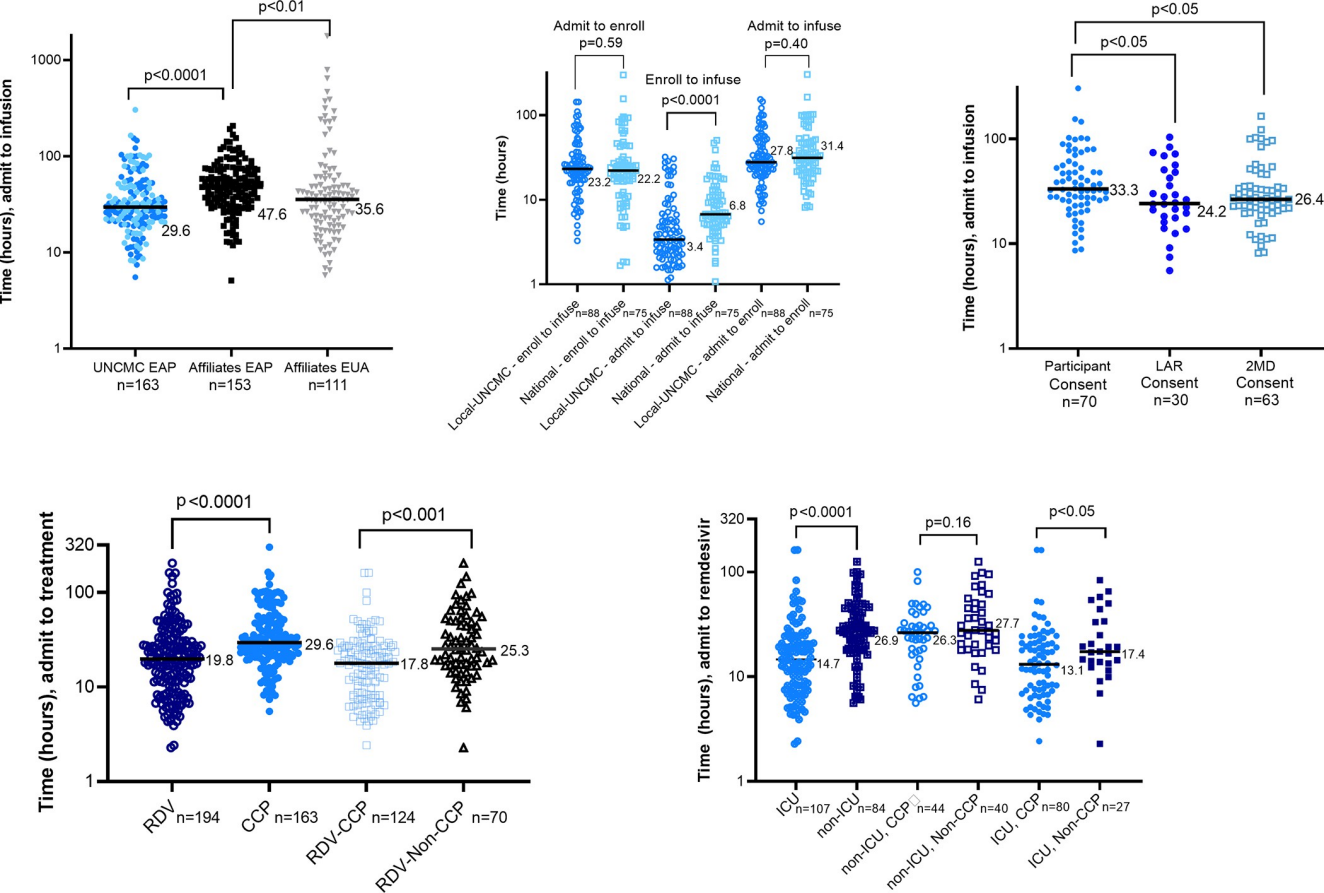
Time to CCP infusion comparisons. (A) Time from admission to CCP infusion of recipients in the UNCMC EAP (blue circles) versus affiliates EAP (black squares) versus affiliates EUA (grey inverted triangles). (B) Time from admission to enrollment, enrollment to CCP infusion and admission to CCP infusion for recipients in the UNCMC EAP comparing those that received CCP units from the UNCMC Blood Donation Center (dark blue open circles) versus a national vendor (light blue open squares). (C) Time from admission to CCP infusion for type of EAP consent obtained at UNCMC (participant consent in light blue circles, LAR consent in dark blue circles, 2MD in open squares). (D) During the CCP EAP era at UNCMC, first comparison is time from admission to remdesivir for all who received remdesivir (dark blue open circles) versus time from admission to CCP infusion of EAP participants (middle blue circles); second comparison is time from admission to remdesivir for all who received remdesivir and CCP (light blue open squares) versus those who received only remdesivir (black open triangles). (E) During the CCP EAP era at UNCMC, time from admission to remdesivir: first comparison is between those that received remdesivir in the ICU (middle blue circles) versus non-ICU (dark blue squares); second comparison is between those that received remdesivir and CCP (middle blue open circles) versus only remdesivir in the non-ICU (dark blue open squares); third comparison is between those that received remdesivir and CCP (middle blue circles) versus only remdesivir in the ICU (dark blue squares). Medians are reported. P values obtained via a non-parametric Mann-Whitney U test.

Affiliate hospitals treated 153 patients with CCP via the EAP (S1 Table). The time from admission to EAP CCP infusion was overall faster at UNCMC than affiliate hospitals, with a median of 29.6 hours (IQR 21.2–48.1) versus 47.6 hours (IQR 32.6–71.6); P<0.0001 (Fig 2A and S2 Fig). Comparing patients receiving CCP at UNCMC to affiliate hospitals, respectively, 84.7% versus 75.8% were treated within 72 hours of admission, 74.8% versus 51% within 48 hours of admission, and 35.0% versus 11.8% within 24 hours of admission.

The majority of the time from admission to infusion was spent performing the aggregate activities of initial evaluation, consultant approval, consent, and enrollment (median 22.6 hours, IQR 16.2–33.0), whereas ~18% of the time (median 5.3 hours, IQR 3.0–9.7) transpired from physician order entry to nursing documented transfusion time. Except for 3 CCP units, during the first 9 weeks of the EAP, UNCMC relied exclusively on CCP that was collected and stored at the UNCMC Blood Donation Center and UNCMC Blood Bank. Subsequently, a combination of on-site CCP and CCP acquired from a national supplier were used (N = 88, 54.0% and N = 75, 46.0%, respectively). The lag-time between national supplier and locally sourced CCP (median 6.8 hours (IQR 5.1–11.8) vs 3.4 hours (IQR 2.3–6.5) (P<0.0001) was not sufficient to significantly decrease total time to infusion after admission (Fig 2B).

The time from admission to CCP infusion for participants consented directly was slower than other modes of consent (Fig 2C). There was no significant difference in time from admission to CCP infusion by consent language (S3A Fig). The only patient-specific factor that discriminated time from admission to CCP infusion among CCP recipients was ABO type, with longer delays for the less common and less available B blood type (median 36.0 hours, IQR 26.9–68.8 vs 31.8 hours, IQR 20.8–46.6, type A and 27.7 hours, IQR 19.5–47.6, type O; p <0.05) (S3B–S3F Fig).

Remdesivir was directly available through the pharmacy with ID approval during this time period, and did not require additional processes for consent, enrollment and communication with the UNCMC Blood Bank. Using time to remdesivir as a comparator, it was estimated that there was an additional time burden of ~9.8 hours (Fig 2D) associated with the receipt of CCP. However, compared with non-CCP recipients, CCP recipients experienced faster time to remdesivir (Fig 2D)–primarily in the ICU where time to remdesivir was faster than the non-ICU COVID-19 care unit (Fig 2E).

### Adoption

Of 11 hospitals in UNC Health, 5 participated in the EAP (S1 Table), and UNCMC served as an early access point for 6 hospitals unable to provide CCP. There was a 17-day lag between the first CCP recipient at UNCMC versus an affiliate hospital.

**Fig 3:**
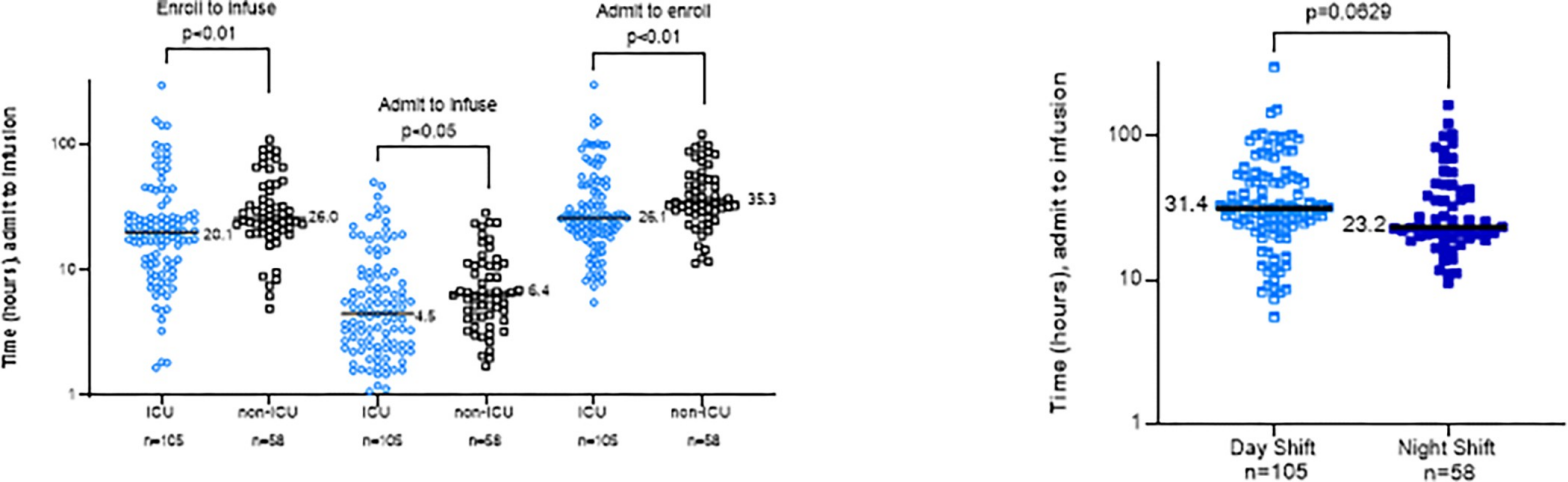
Time to CCP infusion comparisons. (A). Time from admission to enrollment, enrollment to CCP infusion and admission to CCP infusion via the EAP at UNCMC in the ICU (blue open circles) versus the non-ICU (black open squares). (B) Time from admission to CCP infusion via the EAP at UNCMC for those admitted during the day shift (7am-7pm) (middle blue open squares) versus the night shift (7pm-7am) (dark blue squares). Medians are reported. P values obtained via a non-parametric Mann-Whitney U test.

At UNCMC, both the ICU and non-ICU COVID-19 units enrolled participants. One hundred and five (64.4%) CCP recipients required medical ICU-level of care at the time of CCP transfusion (Table 3). Both the time from admission to enrollment and enrollment to infusion were faster in the ICU (Fig 3A).

**Table 3:** CCP recipient characteristics.

		Total (n = 163*)	April (n = 7*)	May (n = 30*)	June (n = 50*)	July (n = 48*)	August (n = 28*)	p-value
**Admissions (including non-recipients) per month, n (%)**		163 (31.0) (n = 526)	7 (21.9) (n = 32)	30 (30.3) (n = 99)	50 (41.0) (n = 122)	48 (32.7) (n = 147)	28 (22.2) (n = 126)	0.199
**Covid-care unit location at time of 1**^**st**^ **infusion, n (%)**	ICU	105 (64.4)	6 (85.7)	25 (83.3)	31 (62.0)	24 (50.0)	19 (67.9)	0.001
	non-ICU	58 (35.6)	1 (14.2)	5 (16.6)	19 (38.0)	24 (50.0)	9 (32.1)	
**Type of consent, n (%)**	Participant	70 (42.9)	5 (71.4)	11 (36.6)	23 (46.0)	16 (33.3)	14 (50.0)	0.031
	LAR	30 (18.4)	2 (28.6)	13 (43.3)	7 (14.0)	5 (10.4)	4 (33.0)	
	2MD	63 (38.7)	0 (0)	6 (20.0)	20 (40.0)	27 (56.3)	10 (35.7)	
**Spanish Interpreter for consent, n (%)**		36 (26.5)	5 (71.4)	10 (33.3)	10 (20.0)	7 (14.6)	4 (14.3)	0.545
**Enrollment day, n (%)**	Weekday	125 (76.7)	7 (100)	25 (83.3)	39 (78.0)	34 (70.8)	20 (71.2)	0.367
	Weekend	38 (23.3)	0 (0)	5 (16.6)	11 (22.0)	14 (29.2)	8 (28.6)	
**Days from symptom onset to 1**^**st**^ **infusion, median (IQR)**		8.0 (6.0–11.0) (n = 158)	8.0 (2)	7.5 (6)	8.0 (4) (n = 49)	9.5 (5) (n = 44)	8.5 (4)	0.405
**Days from diagnosis to 1**^**st**^ **infusion, median (IQR)**		4 (1.0–6.5)	4.0 (4)	3.5 (5)	3.0 (5)	4.0 (6.5)	5.5 (5)	0.127
**OSH Transfer, n (%)**		65 (39.9)	4 (57.1)	16 (53.3)	16 (32.0)	17 (35.4)	12 (42.9)	0.298
**Eligibility Criteria met, n (%)**	High risk of progression to severe or critical disease**	10 (6.2) (n = 161)	0 (0)	3 (10.0)	3 (6.1) (n = 49)	3 (6.3)	1 (3.7) (n = 27)	0.829
	Severe or critical disease***	151 (93.8) (n = 161)	7 (100)	27 (90.0)	46 (93.9) (n = 49)	45 (93.8)	26 (96.3) (n = 27)	
**Respiratory support at time of 1**^**st**^ **infusion, n (%)**	MV	34 (20.9)	1 (14.2)	8 (26.7)	11 (22.0)	9 (18.6)	5 (17.9)	0.247
	HFNC, BiPAP, CPAP, or ≥7L/min NC	64 (39.3)	4 (57.1)	15 (50.0)	19 (38.0)	14 (9.2)	12 (42.9)	
	LFNC (≤ 6L/min NC)	50 (30.7)	2 (28.6)	6 (20.0)	14 (28.0)	18 (37.5)	10 (35.7)	
	RA	15 (9.2)	0 (0)	1 (3.3)	6 (12.0)	7 (14.6)	1 (3.6)	
**Received 2 units, n (%)**		143 (87.7)	5 (71.4)	21 (70.0)	46 (92.0)	46 (95.3)	25 (89.3)	0.007
**Individuals who received UNCMC units vs ARC units, n (%)**	UNC Units	88 (54.0)	7 (100)	28 (93.3)	35 (70.0)	14 (29.2)	4 (14.3)	<0.001
	ARC Units	75 (46.0)	0 (0)	2 (6.67)	15 (30.0)	34 (70.8)	24 (85.7)	
**Time from Admit to 1**^**st**^ **infusion, n (%)**	<24 hrs	57 (35.0)	1 (14.3)	9 (30.0)	21 (42.0)	14 (29.2)	12 (42.9)	0.105
	24–48 hrs	65 (39.9)	1 (14.3)	13 (43.3)	22 (44.0)	18 (37.5)	11 (39.3)	
	48–72 hrs	16 (9.8)	3 (42.9)	1 (3.3)	3 (6.0)	7 (14.6)	2 (7.1)	
	>72 hrs	25 (15.3)	2 (28.6)	7 (23.3)	4 (8.0)	9 (18.8)	3 (10.7)	

*unless otherwise noted

**deemed by treating provider

***Patient met one or more of the following criteria: dyspnea, respiratory rate ≥30/min, SpO2 ≤93%, PaO2/FiO2<300, lung infiltrates >50% within 24–48 hours, respiratory failure, septic shock, multiple organ dysfunction or failure

N = number, ARC = American Red Cross, IQR = interquartile range, ICU = intensive care unit, LAR = legally authorized representative, 2MD = 2 medical doctors, OSH = outside hospital, MV = mechanical ventilation, HFNC = high flow nasal cannula, BiPAP = bilevel positive airway pressure, CPAP = continuous positive airway pressure, NC = nasal cannula, LFNC = low flow nasal cannula, min = minute, RA = room air, hrs = hours, ARC = American Red Cross

Neither time of admission (i.e. night shift (7:00 PM—6:59 AM) (N = 58) vs day shift (7:00 AM– 6:59 PM) (N = 105) (Fig 3B)) nor admission on weekend versus weekday affected time to enrollment or infusion.

### Implementation

The UNCMC CCP program was a clinical translational research program co-led by two ID physician scientists consisting of three primary domains: a clinical domain (CCP collection and infusion of CCP under the EAP or RCT protocols), a virology-immunology domain (processing donor plasma for quantification of nAb), and a data administrative domain (entry and management of participant and program data and reporting to sponsors). Each domain was led by one or two faculty co-investigators. The organizational structure remained stable throughout the program. Collectively, a total of 14 key personnel contributed to program activities. All but one temporary position were existing UNCMC employees who re-deployed their efforts to the program when their pre-pandemic clinical and research activities were paused. Scientific, clinical, and operational advisors guided the inception and ongoing implementation of the program. The clinical treatment team was available at all times, including weekends, and consisted of an on-call ID physician (rotating between 5 MDs) and up to two study coordinators (rotating between 1 MD, and 2 NPs) (Fig 1).

Of 925 individuals contacted for donation, transfusable CCP units were collected from 127 (13.7%) unique volunteers over 22 collection dates. Thirty-two individuals donated at least twice resulting in a total of 170 donations and 449 transfusable plasma units collected. Eighty-seven of 163 recipients (53.4%) received their CCP units from UNCMC plasma bank.

In accordance with emerging guidelines and data, on June 17, 2020, the CCP team discouraged use of CCP in patients on mechanical ventilation for >3 days and those with symptoms onset >10 days prior to admission. After the first two months of the program, the proportion of non-ICU to ICU patients increased and then remained stable. The median time from diagnosis and symptoms onset to infusion did not decrease over time (Table 3).

### Maintenance

The program was implemented and operated stably over approximately 5 months. The proportion of admitted patients receiving CCP ranged from 21.9% (month 1) to a peak of 41.0% (month 3) as cases fluctuated over time (Table 3).

**Fig 4:**
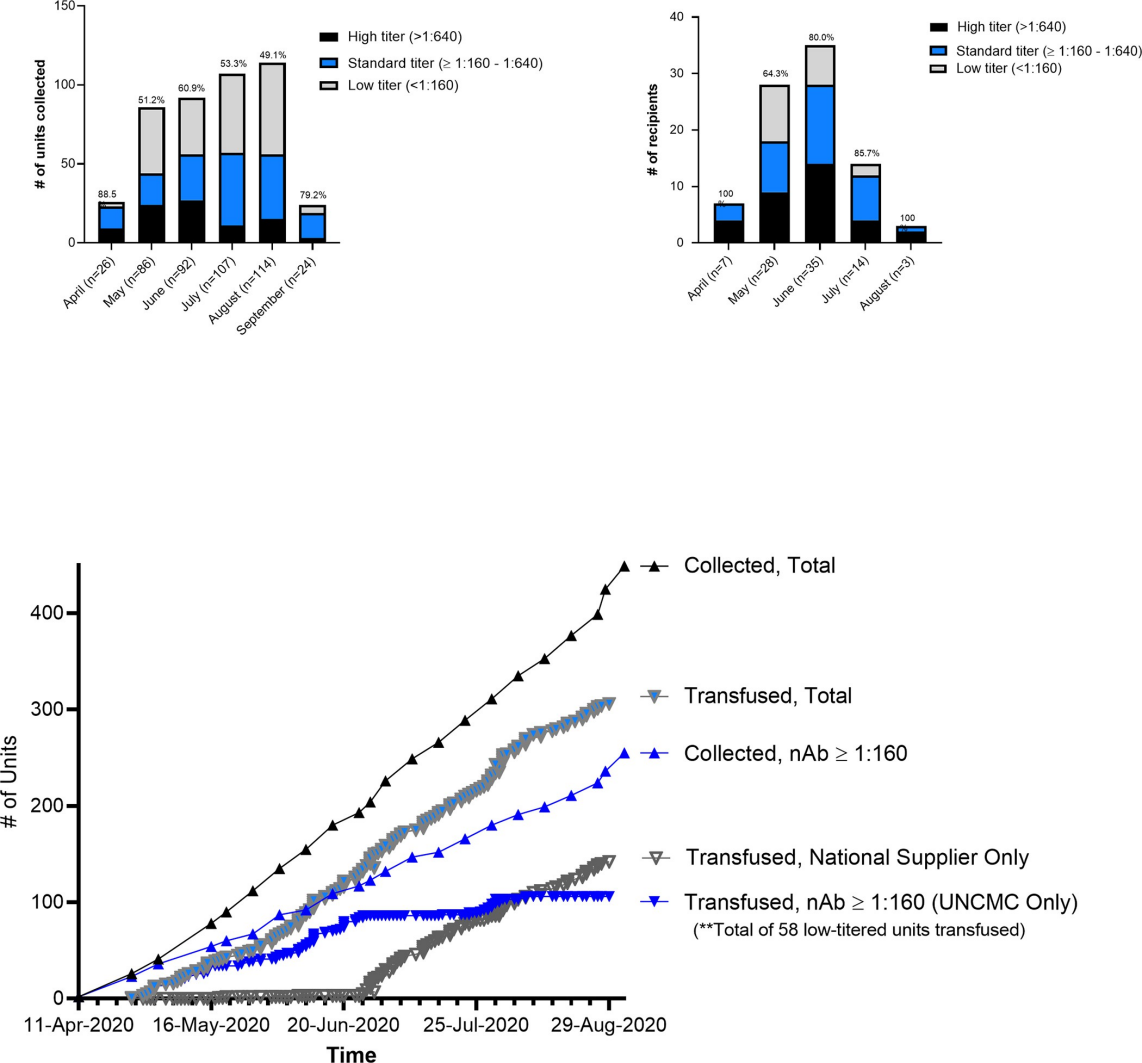
Proportions and cumulative titered units collected and transfused during the EAP era at UNCMC. (A) Monthly proportions of low (gray) versus standard (blue) versus high (black) titered units collected. Percentages at tops of bars are cumulative proportions of standard + high titered units. (B) Monthly proportions of low (gray) versus standard (blue) versus high (black) titered units transfused. Percentages at tops of bars are cumulative proportions of standard + high titered units. (C) Cumulative total units collected and transfused over time, cumulative units with titers ≥1:160 collected and transfused over time, cumulative national supplier units transfused over time.

The program collected a median of 22 transfusable units from approximately 8 donors per week. The research laboratory performed nAb titers, although inpatient CCP needs outpaced turnaround time required for nAb titers challenging provision of real-time results. Further, despite efforts to target donors more likely to generate higher nAb titers, the proportion of CCP with titers <1:160 did not decrease over time (Fig 4A and 4C). Overall, 68 out of the 87 (78.2%) UNCMC CCP recipients that received CCP with known titers, received a nAb titer ≥1:160 and 73 (83.9%) >1:80 (Fig 4B and 4C).

**Fig 5:**
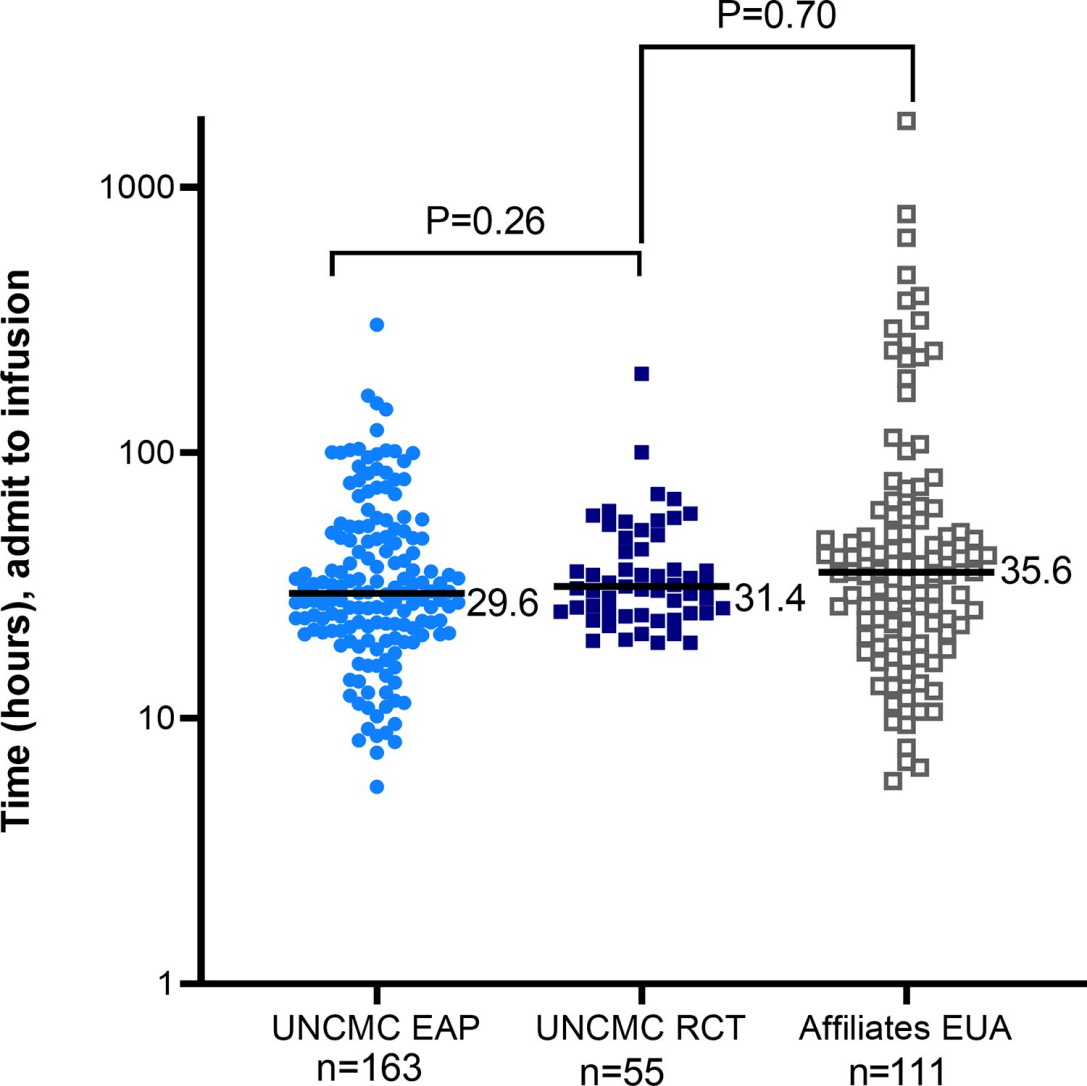
Time from admission to CCP infusion of EAP and RCT recipients at UNCMC and of EUA recipients at affiliates. UNCMC EAP reported in middle blue circles, UNCMC RCT reported in dark blue squares, Affiliates EUA reported in gray open squares. Medians are reported. P values obtained via a non-parametric Mann-Whitney U test.

As the EAP closed, transition to administering CCP primarily through UNCMC’s RCT was seamless within the structure developed during the EAP. Among 55 RCT participants, all received CCP with nAb ≥ 1:160 within 10 days of symptoms onset, and the RCT did not significantly delay time from admission to infusion (Fig 5). The RCT also provided CCP infusion in a comparably rapid time frame, compared to contemporaneous patients receiving CCP by the EUA mechanism used at affiliates (Fig 5).

## Discussion

A comprehensive summary of the EAP for CCP [12] as well as the serological repertoire and clinical efficacy of CCP across a spectrum of COVID-19 disease severity, including its role among patients with immunosuppression, has been described previously [29–44]. Expert opinion suggests that if CCP has a mortality benefit in COVID-19 treatment, infusion needs to be given early in the disease course, and the CCP needs an adequate concentration of nAb [30, 31, 37, 40]. The novelty of our study, however, is a comprehensive evaluation of a local CCP program, including a description of the core elements and personnel required to implement such a program during a pandemic.

Overall, our evaluation suggests that the UNCMC CCP program achieved some impact based on metrics following RE-AIM, having reached many COVID-19 inpatients, including those members of racial and ethnic minority groups disproportionately affected by COVID-19 [45], and having rapidly provided CCP with FDA-recommended minimum-titer to most patients in ICU and non-ICU units, although time to infusion was shorter in the ICU. Our program required substantial resourcing and staffing for implementation, but was maintained whether administered through a compassionate use mechanism or an RCT. Our experience demonstrates that existing blood collection infrastructure at a single institution can be leveraged during a global pandemic to rapidly pivot to collecting and infusing convalescent plasma for a novel infectious agent, and also suggests that access to a national CCP pipeline may be helpful to supplement local collection efforts.

Within the first weeks of COVID-19 emerging in our catchment area, it became apparent that a dedicated CCP team would be needed to achieve our goal of reaching as many eligible patients as possible. The team overcame several implementation barriers, including fulfilling regulatory and reporting requirements for emergency investigational therapy use; efficiently using scarce personal-protective equipment for consent, laboratory testing, and infusion; and rapidly deploying regulatory-approved multi-lingual mobile e-consent tools. The CCP team integrated safely, ethically, and efficiently into existing COVID-19 clinical workflows without obstructing routine operations in the ICUs and wards.

Almost all patients approached by the study team completed enrollment and all those enrolled received CCP infusion. COVID-19 risk factors and disease severity differed by CCP receipt type, as expected, but demographic characteristics were comparable between CCP recipients and non-recipients. Timing of CCP infusion differed by admitting ward and patient blood type, again as expected, but not by consent process or need for Spanish-language interpreter.

A major success of our program was our ability to deliver CCP to patients in just over one day from admission. While sourcing plasma from our on-site bank was slightly faster than ordering from a nearby national supplier, our ability to more rapidly infuse CCP compared to our affiliate hospitals is likely due to the additional core personnel and resources dedicated to referral and enrollment into the UNCMC program. Indeed, the less burdensome demands on providers involved with the streamlined and later to emerge EUA process closed the infusion time gap at affiliate hospitals. Within our medical center, differences in the care team structure, mode of consent, patient acuity, nurse-to-patient ratios, and process familiarity (like routine type and screen and general blood transfusion consent) likely contributed to more rapid infusion times in the ICU.

Some data supports greater CCP effectiveness at higher nAb titers, with some suggesting therapeutic titer levels >1:640 [30, 31, 37, 46, 47]. Through our local CCP program, a high proportion of infused CCP contained at least the FDA-recommended minimum nAb titer of 1:160—approximately half of which contained high nAb titer (1:640) CCP. While these proportions are higher than those reported nationally [37], like other academic centers [13], we struggled to fill our CCP bank with high-nAb titer CCP. Despite persistent recruitment efforts at UNCMC to sustain a sufficient quantity of CCP, only a small proportion of contacts (13.7%) presented for voluntary donation. Further, despite efforts to target individuals likely to have high-titer plasma, we, like most donation centers [48], had to rely on post-collection assays to qualify CCP. Consequently, our bank became disproportionately filled with low nAb titer CCP. This key deficiency in the program highlights the need for future efforts to provide more resources to rapidly develop, expedite, and distribute reliable antibody assays to pre-qualify donors before expending unnecessary resources on low-nAb titer CCP, or perhaps highlights the opportunity for future advancements in methods to concentrate and pool low-nAb titer CCP. Other areas for improvement in the program included ensuring universal assessment for CCP eligibility among inpatients and providing CCP infusion within 10 days of symptom onset for consenting patients. The latter primarily represented patients who were late presenting for care and who did so before outpatient therapeutics were available.

At inception, one of our program’s goals was to conduct a CCP treatment RCT as quickly as possible. In practice, we gave more CCP via compassionate use EAP than through the RCT. This imbalance resulted from our inability to stockpile an adequate supply of nAb-titered defined CCP before our hospital met a rapid influx of admissions for COVID-19, and our concerns that integrating the RCT may create an access barrier to rapid treatment for patients. When the FDA replaced the EAP with the EUA mechanism, we were able to seamlessly transition instead to the RCT. Despite the more complicated clinical trial protocol, infusion times for clinical trial participants were similar to EAP participants at UNCMC and no different from infusion times for contemporaneous EUA participants at affiliate hospitals. These observations demonstrate that operating clinical trials in acute care settings, even during a pandemic response, may not interfere or delay routine care. Rather, we found that CCP recipients received remdesivir faster than non-recipients and were more likely to receive other COVID-19 specific therapies, suggesting that the program did not have unintended negative effects on receiving COVID-19 treatment, which is a key consideration in the RE-AIM definition of effectiveness [15, 17].

Implementation evaluations typically focus on understanding the adoption and integration of therapies with proven clinical efficacy into routine health systems. However, in the context of the novel, rapidly spreading and fatal SARS-CoV-2 virus, there was an unprecedented reversal of this typical sequence: the nationally endorsed putative therapy, CCP, was widely distributed before consensus of clinical effectiveness was obtained. We therefore applied a broader definition of implementation effectiveness, patterned after the RE-AIM framework [15] and consistent with literature focused on understanding how to introduce potential solutions into the health system [49, 50], with the intention of critically examining unprecedented implementation issues that newly emerged with COVID-19 [51]. We believe that the question of ‘how’ to roll out emergent therapeutics, even prior to proven clinical efficacy, during a pandemic is an important one. Indeed, evaluations of our program, and programs like it, are essential for guiding preparation and rational use of resources for ongoing and future pandemics.

Our findings should provide trialists and patients at academic medical centers with confidence that participating in a clinical trial does not impede routine care. Thus, as noted by others after the 2014–2015 West African Ebola epidemic [20, 21], it seems worthwhile to coordinate response efforts early during future pandemics at centers that have the capacity to quickly collect, qualify, and infuse titer-defined convalescent plasma in multi-center RCTs.

In summary, we successfully and rapidly implemented and maintained a local CCP collection, treatment, and clinical trial program at a single highly resourced academic medical center. We found that a dedicated CCP team comprised of personnel from ID and clinical trial units could be re-deployed to appropriately reach the intended population of COVID-19 patients with timely infusion of titered CCP. The greatest barrier we encountered to CCP program implementation was collecting adequate supply of high-titer plasma. We hope our findings prompt critical reflection, planning, and consideration of including a dedicated therapeutics and convalescent plasma team as part of future integrated pandemic response efforts.

## Supporting information

S1 Fig CCP program timeline. Functional neutralizing antibody assays were available by April 1, 2020 and assay results from CCP donors were reported on a rolling basis every 2–4 weeks throughout 12/01/2020.
(TIFF)

S2 Fig Time from admission to CCP infusion of EAP and EUA recipients at UNCMC and affiliates. Medians are reported. P values obtained via a non-parametric Mann-Whitney U test.
(TIF)

S3 Fig Comparisons of recipient factors of time from admission to CCP infusion of EAP participants at UNCMC. (A) those that were consented in English vs those that required use of a Spanish interpreter, (B) sex, (C) race, (D) ethnicity, (E) age, and (F) blood type. Medians are reported. P values obtained via a non-parametric Mann-Whitney U test.
(TIF)

S1 Table UNC health system hospitals that participated in the CCP EAP and EUA. (TIFF)
