# Supplementary figures and images for: Evaluation of a COVID-19 convalescent plasma program at a U.S. academic medical center
Source: PLoS One. 2022 Dec 8;17(12):e0277707. doi: 10.1371/journal.pone.0277707 (PMC9731422; doi:10.1371/journal.pone.0277707)

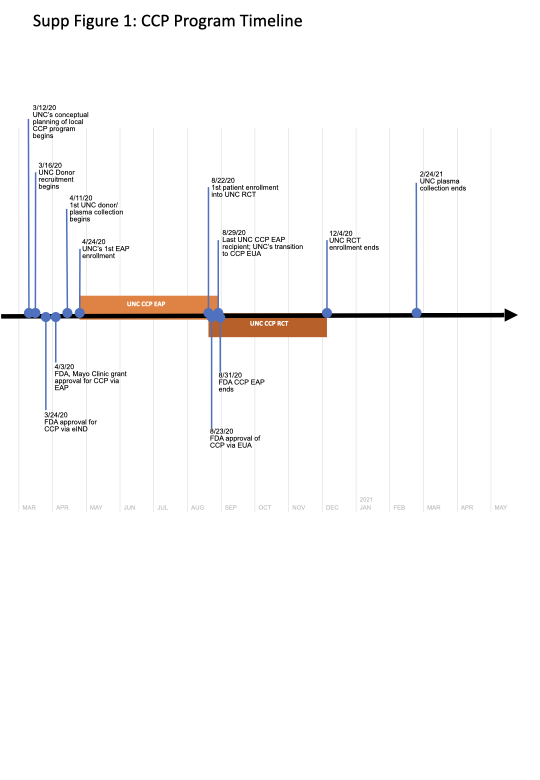

Supplement: S1 Fig — Functional neutralizing antibody assays were available by April 1, 2020 and assay results from CCP donors were reported on a rolling basis every 2–4 weeks throughout 12/01/2020. (TIFF) [file pone.0277707.s001.tiff]

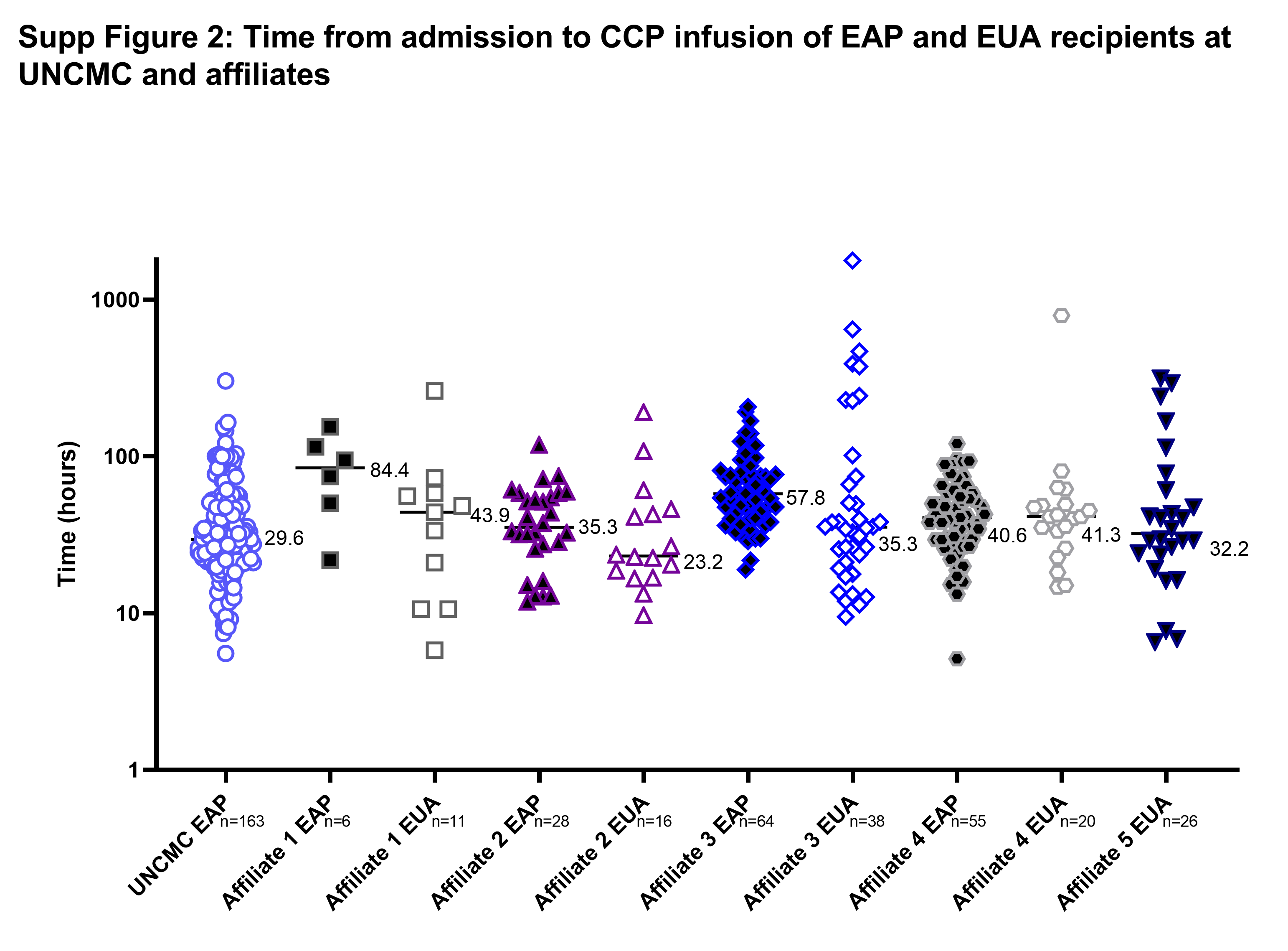

Supplement: S2 Fig — Medians are reported. P values obtained via a non-parametric Mann-Whitney U test. (TIF) [file pone.0277707.s002.tif]

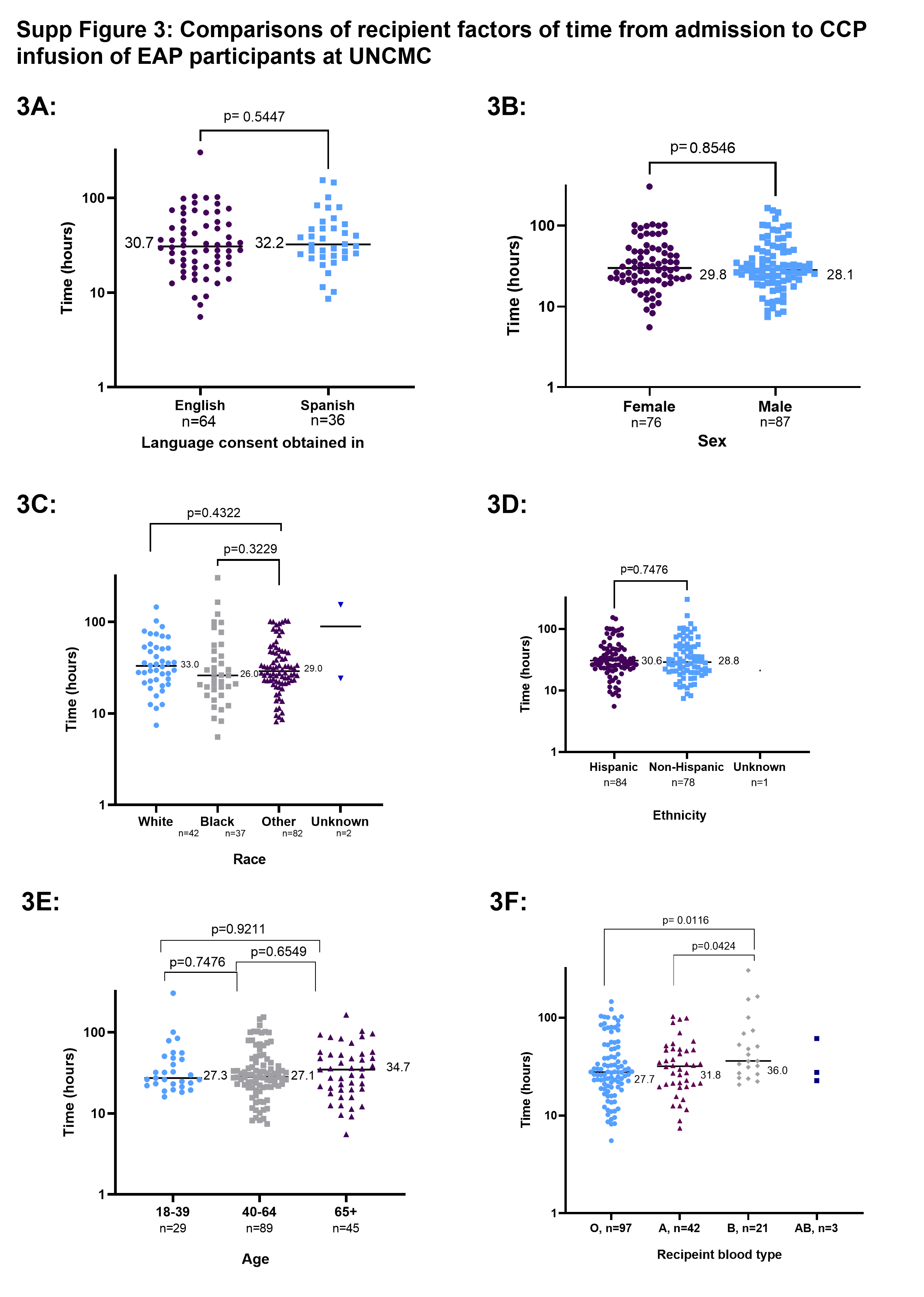

Supplement: S3 Fig — (A) those that were consented in English vs those that required use of a Spanish interpreter, (B) sex, (C) race, (D) ethnicity, (E) age, and (F) blood type. Medians are reported. P values obtained via a non-parametric Mann-Whitney U test. (TIF) [file pone.0277707.s003.tif]

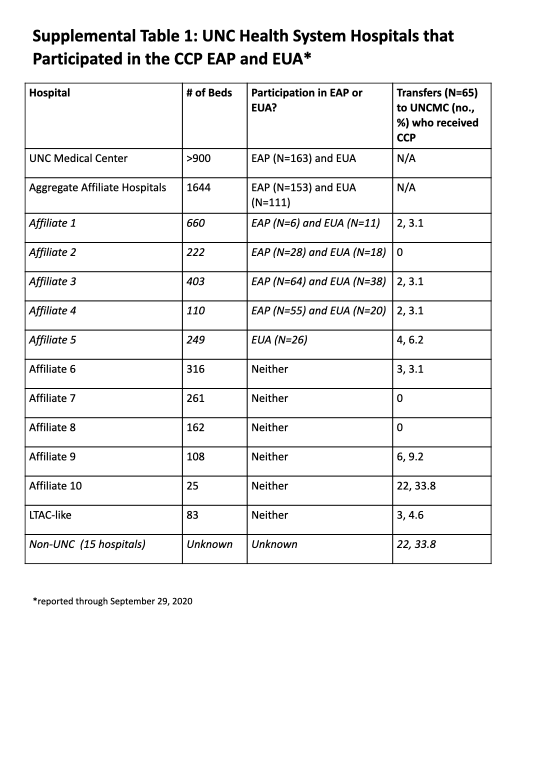

Supplement: S1 Table — (TIFF) [file pone.0277707.s004.tiff]
