# Supplementary material for: Evaluation of a COVID-19 convalescent plasma program at a U.S. academic medical center
Source: PLoS One. 2022 Dec 8;17(12):e0277707. doi: 10.1371/journal.pone.0277707 (PMC9731422; doi:10.1371/journal.pone.0277707)
Supplement: Attachment — Submitted filename: Response to reviewers.docx [file pone.0277707.s005.docx]

We greatly appreciate the invitation to submit a revision of our paper along the lines suggested in the editors’ and reviewers’ comments. The reviewers’ careful, thoughtful and constructive comments have allowed us to greatly improve our revised manuscript.

Below we describe the revisions we made to the manuscript based on recommendations from the editors and reviewers. Editorial and reviewers’ comments are reproduced verbatim in the order of their mention (***in bold, italicized text***), followed by our response. Line numbers in our responses align with respective location in the track changes version of our revised manuscript.

**SUMMARY COMMENTS FROM THE EDITORS**

***Journal Requirements:***

***When submitting your revision, we need you to address these additional requirements.***

***1. Please ensure that your manuscript meets PLOS ONE's style requirements, including those for file naming. The PLOS ONE style templates can be found at 
https://journals.plos.org/plosone/s/file?id=wjVg/PLOSOne_formatting_sample_main_body.pdf and*** 
[***https://journals.plos.org/plosone/s/file?id=ba62/PLOSOne_formatting_sample_title_authors_affiliations.pdf***](https://journals.plos.org/plosone/s/file?id=ba62/PLOSOne_formatting_sample_title_authors_affiliations.pdf)

We have done this.

***2. Please include a link to the trial registry of the related RCT in the methods section of your paper. Thank you.***

Thank you for pointing this out. The following link has been included in the methods section: <https://clinicaltrials.gov/ct2/show/NCT04524507> (See line 121).

***3. Thank you for stating the following in the Competing Interests section:***

***I have read the journal's policy and the authors of this manuscript have the following competing interests: Actavis, Tetraphase, Sanofi-Pasteur, MedImmune, Astellas, Merck, Allergan, T2Biosystems, Roche, Achaogen, Neumedicine, Shionogi, Pfizer, Entasis, QPex, Wellspring, Karius, Utility, Johnson&Johnson, Novartis, Cidara, BSAC, Ridgeback Biopharmaceuticals, Janssen, Syneos***

***Please confirm that this does not alter your adherence to all PLOS ONE policies on sharing data and materials, by including the following statement: "******This does not alter our adherence to  PLOS ONE policies on sharing data and materials.” (as detailed online in our guide for authors http://journals.plos.org/plosone/s/competing-interests).  If there are restrictions on sharing of data and/or materials, please state these. Please note that we cannot proceed with consideration of your article until this information has been declared.***

***Please include your updated Competing Interests statement in your cover letter; we will change the online submission form on your behalf.***

We have added the following statement, “This does not alter our adherence to PLOS ONE policies on sharing data and materials” to our competing interests section in the revised cover letter.

***4. We note that you have included the phrase “data not shown” in your manuscript. Unfortunately, this does not meet our data sharing requirements. PLOS does not permit references to inaccessible data. We require that authors provide all relevant data within the paper, Supporting Information files, or in an acceptable, public repository. Please add a citation to support this phrase or upload the data that corresponds with these findings to a stable repository (such as Figshare or Dryad) and provide and URLs, DOIs, or accession numbers that may be used to access these data. Or, if the data are not a core part of the research being presented in your study, we ask that you remove the phrase that refers to these data.***

We have removed the phrase, “data not shown” as this is not a core part of the research being presented.

**REVIEWER #1**

***In this retrospective study, the authors provide an evaluation of the feasibility of operating a COVID-19 convalescent plasma (CCP) program. Compared to affiliate hospitals the UNC CCP program provided significantly faster CCP infusion after admission. Nearly 80% of CCP recipients received the FDA recommended minimum titer.***

Thank you for your directive comments. We have made all the revisions as suggested.

***Minor revisions:
1- Abstract: Define UNC.***

We have done this.

***2- Abstract: Provide a measure of dispersion for time to CCP infusion for UNC and for affiliate hospitals.***

We have added a measure of dispersion for time to CCP infusion for UNC and for affiliates (see lines 59-60).

***3- Tables: Replace “no.” with “n” since this is the standard statistical abbreviation.***

We have replaced “no.” with “n” in the tables as advised.

***4- Lines 234 and 279: For clarity, identify the test as the Mann-Whitney U test.***

We have clarified the test as the “Mann-Whitney U test” throughout.

***5- Figures: Include numeric p-values instead of NS.***

We have included numeric p-values instead of NS throughout.

**REVIEWER #2**

***This study applies implementation science frameworks in attempt to evaluate the routine use of COVID-19 convalescent plasma (CCP), an intervention with limited evidence of efficacy.

Herein is my primary concern:

"Implementation science (IS) is the study of methods to promote the adoption and integration of evidence-based practices, interventions, and policies into routine health care and public health settings to improve our impact on population health" https://cancercontrol.cancer.gov/is/about

Using implementation science frameworks to routinize an intervention of unproven efficacy goes against the principles and ethos of implementation science.
The authors failed to establish that CCP is an evidence-based intervention which they want to integrate into routine practice. The literature suggests conflicting evidence on the efficacy of CCP. Examples of studies report are as follows:

"CCP therapy did not result in beneficial virological or clinical improvements." (2021)
https://bmjopenrespres.bmj.com/content/8/1/e001017.abstract

"CCP did not meet prespecified outcomes for efficacy, but high-titer CCP may have benefited hospitalized patients with COVID-19 early in the pandemic when other treatments were not in use" (2021) https://jamanetwork.com/journals/jamainternalmedicine/article-abstract/2787090***

***The authors themselves acknowledge this gap in their study when they stated that: "COVID-19 convalescent plasma (CCP) emerged as a leading therapeutic option, although evidence of its efficacy for any infection was limited"

I do acknowledge that interim recommendations were made for use of CCP. E.g.

"Based on the available evidence, AABB developed interim recommendations for CCP use. These interim recommendations will be updated as more peer‐reviewed clinical trial data are published." https://www.ncbi.nlm.nih.gov/pmc/articles/PMC8014606/***

We agree that there remains clinical equipoise surrounding the role of CCP, if any, as a therapeutic for COVID-19. We have added the three references mentioned above to our manuscript (reference #’s 28 - 30). There are many others, as well. Elaborating on the interpretation of the many CCP trials and whether sufficient evidence supports current FDA emergency use and IDSA guidelines recommendations (<https://www.idsociety.org/covid-19-real-time-learning-network/therapeutics-and-interventions/convalescent-plasma/#:~:text=Ambulatory%20Patients%3A%20IDSA%20guidelines%20suggest,no%20other%20treatment%20options%20(conditional>) is beyond the scope of this manuscript. We refer the reviewer to our pre-print <https://www.medrxiv.org/content/10.1101/2022.04.29.22274387v1.full-text> (reference #46) that delineates the importance of defining antibody content in CCP and the limitations of trials that studied CCP without defined antibodies.

***The authors use the RE-AIM framework, however, the indicators used do not align with the intent of the RE-AIM from the literature.

For example, the indicators used to assess "effectiveness" as a RE-AIM domain are "Proportion of patients who received CCP infusion < 72 hours after admission at UNCMC vs affiliate hospitals" and "Time to remdesivir in CCP recipients and non-recipients at UNCMC." These have absolutely nothing to do with "effectiveness". It would seem that the authors are aware that the intervention, not having sufficient evidence of efficacy, may not result in effective outcomes in the real world. Thus, they used a monitoring indicator to replace "effectiveness". In my opinion, this is misleading with respect to the spirit of implementation science.

I believe this article can be re-written as a practice paper from the field, or fit somewhere in the monitoring and evaluation (M&E) literature, but should certainly not be published in the guise of implementation science, as it may project an inappropriate use of implementation science frameworks.***

We appreciate the reviewer’s thoughtful perspective on implementation research, and we are familiar with the NCI definition of “implementation science”. We clarify that in this project we used other, broader, and more inclusive definitions of “implementation research” that have been endorsed by WHO and that we believe are relevant to our study: “Implementation research is the scientific inquiry into questions concerning implementation—the act of carrying an intention into effect, which in health research can be policies, programmes, or individual practices (collectively called interventions).” (please see: *DH Peters et al., BMJ 2013;347:f6753 doi: 10.1136/bmj.f6753*). And from WHO: “Broadly speaking, the term implementation research describes the scientific study of the processes used in the implementation of initiatives as well as the contextual factors that affect these processes. It can address or explore any aspect of implementation, including the factors affecting implementation (such as poverty, geographical remoteness, or traditional beliefs), the processes of implementation themselves (such as distribution of fully-subsidized insecticide-treated bednets (ITNs) through maternal health clinics, or the use of mass vaccination versus surveillance-containment), and the outcomes, or end-products of the implementation under study. Implementation research is applicable and relevant to many different research domains, and to different degrees, depending on the subject under study”. (*see:* [*https://apps.who.int/iris/bitstream/handle/10665/91758/9789241506212_eng.pdf*](https://apps.who.int/iris/bitstream/handle/10665/91758/9789241506212_eng.pdf)).

When we began our program, it was impossible to know what, if any, therapeutics would be effective against the *novel* SARS-CoV-2 virus. Yet, as the first waves of hospitalized patients spread throughout the globe, there was fervent interest in rapidly deploying potential therapeutics. The current literature is almost entirely focused on the clinical outcomes of these various interventions in observational studies and clinical trials. Yet, there is a major gap in the literature regarding objective evaluations of the deployment of these interventions for either emergency use or clinical trial purposes. Indeed, the ability to implement novel interventions and clinical research amidst a rapidly spreading pandemic is a subject of interest in pandemic response literature (see Integrating Clinical Research into Epidemic Response: The Ebola Experience, National Academies of Sciences, Engineering, and Medicine; Health and Medicine Division; Board on Health Sciences Policy; Board on Global Health; Committee on Clinical Trials During the 2014-2015 Ebola Outbreak. Editors: Emily R. Busta, Michelle Mancher, Patricia A. Cuff, Keith McAdam, and Gerald Keusch. Washington (DC): [National Academies Press (US)](http://www.nap.edu/); 2017 Jun 26 ISBN-13: 978-0-309-45776-7). Because we had established *a priori* metrics for delivery of CCP within our program, including through a randomized control trial, we agree with Reviewer #3 that we are in a position to make an important contribution to the literature that we hope can inform future pandemic responses.

That said, we have followed the reviewer’s recommendation to reframe the paper more as a program evaluation and mention that we used a pragmatic application of the RE-AIM framework to that end. We have revised the manuscript title accordingly to **Evaluation of a COVID-19 convalescent plasma program at a U.S. academic medical center**. It is important to note that RE-AIM was designed to be used both for planning and evaluation (see: *Glasgow RE, Estabrooks PE. Pragmatic Applications of RE-AIM for Health Care Initiatives in Community and Clinical Settings. Prev Chronic Dis 2018; 15:170271. DOI: https://doi.org/10.5888/pcd15.170271*.) and has been used as an empirically supported tool for systematic evaluation of a variety of implementation efforts, including real-world clinical programs (please see: *Burke et al. BMC Obesity (2015) 2:21 DOI 10.1186/s40608-015-0050-8, Kwan BM, McGinnes HL, Ory MG, Estabrooks PA, Waxmonsky JA and Glasgow RE (2019) RE-AIM in the Real World: Use of the RE-AIM Framework for Program Planning and Evaluation in Clinical and Community Settings. Front. Public Health 7:345. doi: 10.3389/fpubh.2019.00345*).

These changes in response to the reviewer’s very much appreciated overarching comment about the manuscript are reflected throughout the manuscript (see lines 48-51, 99-102, 151-153).

**REVIEWER #3**

***This manuscript describes a retrospective evaluation of a COVID-19 convalescent plasma (CCP) program implemented at a single highly resourced academic medical center. Although this appears to be a relatively small program (including 163 people who received CCP), these data and associated narrative summary of the CCP program may provide considerations for the use of experimental therapeutics during a pandemic. The investigators report metrics describing the success of the framework of their local program and the performance of their local program, particularly relative to affiliate hospitals in a similar catchment area. This is a novel study with important implications for the next global health calamity.

I have several broad and more specific comments for the authors to consider and/or clarify.***

Thank you for your very thoughtful comments. We very much appreciate both the broad and more detailed comments and have responded to each of them as below. ***Broad Comments***

***1. Because a recent paper was published that appears to provide a large and seemingly comprehensive summary of the Expanded Access Program (EAP) for CCP,1 the novelty of this investigation should be more clearly defined. Notably, the current manuscript adds important information to the literature by describing the local framework that may support operating a local experimental therapeutic program during a pandemic.
1Senefeld JW, Johnson PW, Kunze KL, et al. Access to and safety of COVID-19 convalescent plasma in the United States Expanded Access Program: A national registry study. PLoS Med 2021;18: e1003872. PMID: 34928960.***

Thank you for this suggestion. We have added this reference (reference #12) to the manuscript (see line 422) as well as more clearly defined the novelty of our manuscript (see lines 427-430).

***2. The goals of the program described in the methods do not appear to represent a priori goals, but goals based on new information gleaned during the COVID-19 pandemic. There was limited information on timing of treatment and limited/no available assays to assess neutralizing antibody levels in April 2020. Additionally, the manuscript presents limited information about the ‘successes of these goals, but it could be suggested that these goals were not successfully reached. For example, ~30% of patients received plasma after 10 days of symptom onset, most CCP was provided outside of the local RCT, and many eligible patients did not receive CCP. Consider reframing this section of the manuscript.***

We have clarified in the Methods section (see lines 128-138) which goals were *a priori* program goals and which evolved. We note that the goals for type of titer and timing of treatment evolved quickly as the COVID-19 pandemic evolved after April 2020. In their original guidance statement, the FDA stated “*We recommend neutralizing antibody titers of at least 1:160. A titer of 1:80 may be considered acceptable if an alternative matched unit is not available”* (see Investigational COVID-19 Convalescent Plasma Guidance for Industry, U.S. Department of Health and Human Services, Food and Drug Administration, Center for Biologics Evaluation and Research, April 2020, page 6). Because we had access to neutralizing antibody assays, we did have the goal of a minimum neutralizing antibody titer as an *a priori* goal. We also quickly adopted a goal of 10 days from symptoms onset based on early findings of the kinetics of seroresponse from Wuhan, China and to be consistent with our randomized controlled trial protocol. These goals were incorporated into our randomized controlled trial protocol IND application (IND 22282, submitted May 1, 2020 and issued May 8, 2020). Therefore only 7 participants (Table 3) were treated prior to the intent of treatment within 10 days of symptoms onset. We have added to the Discussion section narrative noting where our program may have fell short in some respects (see lines 485-489), including in delivering CCP late and missing eligible patients, although most of the delay in time from symptom onset to CCP infusion was because of late presentation to care for many patients. In addition, please note that patients could receive CCP outside of the local RCT through our CCP program based on patient and provider preference.

***3. Generally, the methods supporting data curation and presentation are unclear. The authors present comparisons of CCP recipients to patients who received remdesivir. The rationale and methods supporting these comparisons should be more clearly delineated in the methods. The authors focus on CCP associated with the EAP, and (largely) do not report data associated with the local RCT. The authors should provide a justification for removing data associated with the RCT—given that the scope of the manuscript. However, the RCT is discussed at length, and presented in Supplemental material and Figure 5. It is also unclear on how data representing CCP associated with the EUA were collated if data EHR data were censored on 31 August. Additionally, the ethics associated with RCT should likely be presented given that some data are discussed (e.g. Fig 5).***

These are important points that we hope to clarify here. First, we largely focused on the EAP era because we had enough participants and total hospitalizations during this period to have adequate power for comparisons of demographics data and longitudinally. Demographics data from the RCT need to first be reported in the primary publication for the RCT outcomes which is currently under review (<https://www.medrxiv.org/content/10.1101/2022.04.29.22274387v1.full-text> inclusion). Thus, we only report data on the *delivery* infusion times of the RCT recipients here. Since there were only 55 participants during that time period, we also lack adequate power to compare with contemporaneous non-RCT participants. Additionally, the inclusion and exclusion criteria for the RCT required treatment within 10 days of symptoms onset and within 48 hours of hospital admission with a neutralizing antibody titer of at least 1:160. Therefore, by definition, all RCT participants would have met those effectiveness measures. All reported EUA use was from patients at affiliate hospitals. When the EUA replaced the EAP at these hospitals, UNCMC shifted to an RCT. All EUA data from affiliate programs was collated 9/1/2020 and 12/04/2020 to be contemporaneous with the UNCMC RCT era. Lines 140-147 now better clarify these rationale and Table 1 explicitly indicates when primary metrics were focused on the EAP era.

We have added details in lines 235-241 regarding manual extraction of infusion times from the EHR for EUA and RCT enrollees.

We have added an ethics statement for the RCT in the methods section (see line 121).

We used remdesivir as a comparator to attempt to assess whether the CCP program had unintended negative effects on receiving COVID-19 treatment, which is a key consideration in the RE-AIM definition of effectiveness. We have provided this clarification in the methods (see lines 149-150).

***Specific Comments

Lines 62-65: Please clarify this passage — this may be an overstatement or oversimplification. Was the access to or supply of experimental therapeutics limited?***

Both supply of drugs, like remdesivir, and patient access to enroll in pharmaceutical company sponsored RCTs was limited. We have clarified this passage (see lines 72-77).

***Lines 93-98: The ethics statement associated with providing CCP is clearly described. However, the ethics statement associated with collection of data via electronic health records does not appear to be described.***

We have included a statement in the ethics statement in response to this comment (see lines 122-124).

***Table 1, primary metric for reach: The final bullet point includes “10 days of illness onset”, however, most of the text states “symptom(s) onset”. Consider using the same language throughout the manuscript for reader clarity.***

Thank you for drawing attention to this inconsistency. We have changed “illness onset” to “symptoms onset” in Table 1.

***Additionally, it is unclear why CCP relative to symptom onset is a primary metric (as opposed to ‘within 72 hours of admission’, as presented elsewhere). The time between symptom onset and admission does not represent metrics associated with the program, transfusion within 72 hours of admission is likely better represents a metric of the program.***

We have added transfusion within 72 hours of admission as primary metric as a proxy measure for program effectiveness and kept transfusion within 10 days of symptoms onset as an additional primary metric as a more biologically and clinically relevant time-bound measure (see lines 132-133, 137-138). Additionally, we have moved these primary metrics to the effectiveness rather than the reach section (see table 1 and effectiveness section in the results section).

***Lines 187-190: No information about post-hoc testing nor adjustments for multiplicity were provided. Because statistical analyses involved comparisons between two or more groups (e.g., Fig 2C), details about post-hoc testing and/or multiplicity should be addressed.***

We have added to the methods section that in addition to the reported Mann-Whitney tests, we performed one-way ANOVA with multiple comparisons (Tukey-Kramer) (lines 245-247). Following a Tukey-Kramer post-hoc test, none of the p-values were significant for Figure 2C.

***Lines 198-200: The comparator group is not defined. “CCP recipients were more likely to…” compared to whom? According to Table 2, there were no group-related differences in comorbid conditions (P=0.156) between non-CCP recipients and CCP EAP recipients.***

We have clarified this (see line 256-257).

***Line 203: Please provide associated statistics to support the described group-related differences in receiving remdesivir and dexamethasone.***

We have provided these statistics (see Table 2).

***Table 2. Gender and sex are different constructs and likely should not be used interchangeably.***

Thank you for calling attention to this inconsistency. We have changed terminology to “sex” only throughout the manuscript (see Table 2, and S3 Fig supporting information captions).

***Line 224: The term “on-site bank” should include “blood”, or specifically refer to UNC Blood Donation Center.***

We have changed this to “UNCMC Blood Donation Center” throughout the manuscript.

***Line 227: The term ‘EAP recipients’ is not idiomatic. Consider an alternative phrase.***

We have changed this to “EAP participants.”

***Line 237-244: IQR should be defined using the quartile values (as the authors do elsewhere), as opposed to one number.***

We have fixed this (see lines 311-312, 317-318, 324-325).

***Line 245-250: For clarity, the authors should consider referring to ‘CCP infusion’ rather than simply ‘infusion’.***

We have made this change throughout the manuscript.

***Line 240: The authors should more clearly define CCP that is described as “on-site stored CCP”. Perhaps it would be clearer to describe the collection center than the storage center. Is this CCP collected at the UNC Blood Donation Center?***

We have clarified this (see lines 314-315).

***Line 253: Does ‘Blood Bank’ refer to UNC Blood Donation Center?***

We have changed this to, “UNCMC Blood Bank.” (see line 329).

***Line 272: The authors should provide justification for removing April from these analyses while including April in other analyses and visuals.***

We have redone these analyses to include April (see Table 3).

***Line 273: Many abbreviations associated with the table are not defined (e.g. MV, AMC, RA, HFNC).***

We have added the definitions of the abbreviations (see lines 349-353).

***Line 315: “Overall, 68 (78.2%) UNCMC CCP recipients…” The denominator associated with the percent is not clear. It appears that the denominator is 77, which is not found elsewhere in the manuscript.***

We clarified this to the following, “Overall, 68 out of the 87 (78.2%) UNCMC CCP recipients that received CCP with known titers, received …” (see lines 396-397).

***Line 327: The authors do not provide details on collection of data associated with the RCT.***

We have added these details to the Ethics statement (see lines 120-121).

***Lines 340-342: This sentence should be reconsidered. First, expert opinion primarily debates whether or not CCP has a mortality benefit rather than whether or not CCP has a role. Second, the authors fail to mention that CCP has a large role among patients with immunosuppression.***

We have revised this sentence and added a sentence and references #41 and #43 to include information about CCP’s role in immunocompromised patients (see line 422-427).

***Lines 346-349: “including those disproportionately affected by COVID-19”. Where is this statement evidenced? Can the authors clarify?***

We have clarified this statement and added new reference #44 (see lines 433-434).

***Line 351-353: The data presented also suggest that the National pipeline may be helpful to supplement local collection efforts.***

We have added that comment (see lines 441-442).

***Line 364-366: This passage appears to rely on conjecture rather than data presented in the manuscript. Please consider removing or providing supporting evidence.***

We have removed the passage of concern.

***Line 390-393: It may be a reductionist viewpoint to suggest that unnecessary resources are expended on low-nAb titer CCP. Perhaps this highlights the opportunity for future advancements in methods to concentrate and pool low-nAb titer CCP.***

We have added that comment (see lines 484-485).

***Reference 32: This preprint may now be published.2
2Salazar MR, Gonzalez SE, Regairaz L, et al. Risk factors for COVID-19 mortality: The effect of convalescent plasma administration. PLoS One 2021;16: e0250386. PMID: 33914780.***

We have updated this reference to the published version.

***Generic comments on figures: 1) consider providing more descriptive y-axis labels on figures, 2) please define abbreviations in the legends, 3) the authors should consider including precise p-values unless <0.0001, 4) consider using different symbols for different plots and/or colors that include a more color blind-friendly palette (several plots are difficult to visually distinguish)***

We have provided more descriptive y-axis labels on figures, have defined abbreviations in the legends, and included more precise p-values unless <0.0001. Several changes were made to symbols and colors on the figures that are intended to be a more color blind-friendly palette. New co-author, Bridget Nelson, contributed to these figure revisions.

***Figure 1: Please see that “hRS” is written, perhaps the authors intended “hrs”.***

This change has been made.

***Figure 2: The rationale for two different colors associated with UNCMC EAP in Panel A is unclear. Consider including an x-axis title on panel C.***

The rationale for two different colors with UNCMC EAP is because Fig 2B then further splits that group into Local UNCMC CCP units and ARC CCP units. We have defined this more clearly in the legend.

***Figure 5: The abbreviation AMC is not defined. Additionally, several data appear to be outliers. (even on a semi-log scale). Did the authors perform an analysis to distinguish outliers? The authors appear to be presenting data suggesting that one patient had to wait ~6 weeks for a CCP unit.***

We have changed AMC to UNCMC throughout the manuscript.

Yes, we examined for outliers and have double checked our data set for accuracy. Using a ROUT test with Q=1% which identified a total of 39 outliers across all reported categories. When we removed the outliers, the respective p-values uniformly decreased. Since the removal of outliers did not result in loss of significance, we chose to report all raw data with analyses inclusive of the entire data set, indicative of the real-world range of values. All p-values remain as previously reported. Regarding the specific example of a patient waiting multiple weeks for CCP, our center, like others, experienced situations where-in patients, especially those with high-risk factors like immunocompromise, experienced secondary worsening of symptoms resulting in prolonged hospitalizations and/or acquired COVID-19 while inpatient. In these scenarios, treating providers could request CCP as a salvage therapy or for treatment of newly acquired symptomatic COVID-19. New co-author, Bridget Nelson, contributed to these repeat analyses.

***S1 Figure: Consider adding the timeline associated with on-site novel assays to directly measure the functional anti-viral activity of CCP is not depicted.***

We struggled to depict the rolling results from these assays on the figure. We have therefore added to the figure legend: Functional neutralizing antibody assays were available by April 1, 2020 and assay results from CCP donors were reported on a rolling basis every 2-4 weeks throughout 12/01/2020.

***S2 Figure: It is unclear why EAP and EUA data are presented separately and compared to UNCMC EAP. If data associated with UNCMC are restricted to CCP units associated with EAP, it is unclear why data for affiliates would have broader inclusion criteria and incorporate CCP units associated with EUA.***

We understand how this is confusing. We do not report EUA recipients from UNCMC because we prioritized the RCT mechanism for CCP administration rather than the EUA. To further clarify, there was no temporal overlap between the EAP and the EUA. On 8/22/2020 the FDA announced a transition from EAP to EUA. At UNCMC, when the EAP ceased, we routed eligible patients into the RCT. EUA-treated patients at UNCMC were too few to report. Thus, the comparisons between EAP eras at UNCMC and affiliate hospitals are contemporaneous. Likewise, comparisons between RCT at UNCMC and EUA at affiliate hospitals are contemporaneous. This figure therefore compares both the contemporaneous EAP era times to infusion between UNCMC and affiliate hospitals as well as the time to infusion between EAP and EUA eras at the affiliate hospitals.

***While revising your submission, please upload your figure files to the Preflight Analysis and Conversion Engine (PACE) digital diagnostic tool,*** [***https://pacev2.apexcovantage.com/***](https://nam04.safelinks.protection.outlook.com/?url=https%3A%2F%2Fpacev2.apexcovantage.com%2F&data=05%7C01%7Chroot%40montefiore.org%7Cdd19001aa1e345823b4e08da2884c432%7C9c01f0fd65e040c089a82dfd51e62025%7C0%7C0%7C637866848067170539%7CUnknown%7CTWFpbGZsb3d8eyJWIjoiMC4wLjAwMDAiLCJQIjoiV2luMzIiLCJBTiI6Ik1haWwiLCJXVCI6Mn0%3D%7C3000%7C%7C%7C&sdata=HoGGQo06sA2GYW3KCou1yIYuUNKer%2BKAM%2F10ojGRs6Q%3D&reserved=0)***. PACE helps ensure that figures meet PLOS requirements. To use PACE, you must first register as a user. Registration is free. Then, login and navigate to the UPLOAD tab, where you will find detailed instructions on how to use the tool. If you encounter any issues or have any questions when using PACE, please email PLOS at*** [***figures@plos.org***](mailto:figures@plos.org)***. Please note that Supporting Information files do not need this step.***

We have done this.
