# Supplementary material for: Evaluation of a COVID-19 convalescent plasma program at a U.S. academic medical center
Source: PLoS One. 2022 Dec 8;17(12):e0277707. doi: 10.1371/journal.pone.0277707 (PMC9731422; doi:10.1371/journal.pone.0277707)
Supplement: Attachment — Submitted filename: Response to Reviewers_2.docx [file pone.0277707.s006.docx]

We greatly appreciate the invitation to submit another revision of our paper along the lines suggested in the editors’ and reviewers’ comments. The reviewers’ careful, thoughtful and constructive comments have allowed us to greatly improve our revised manuscript.

Below we describe the revisions we made to the manuscript based on recommendations from the editors and reviewers. Editorial and reviewers’ comments are reproduced verbatim in the order of their mention (***in bold, italicized text***), followed by our response. Line numbers in our responses align with respective location in the track changes version of our revised manuscript.

***Journal Requirements:

Please review your reference list to ensure that it is complete and correct. If you have cited papers that have been retracted, please include the rationale for doing so in the manuscript text, or remove these references and replace them with relevant current references. Any changes to the reference list should be mentioned in the rebuttal letter that accompanies your revised manuscript. If you need to cite a retracted article, indicate the article’s retracted status in the References list and also include a citation and full reference for the retraction notice.***

Reference updates include #25 and #47. Both are now published, and the citations have been updated accordingly.

***Review Comments to the Author

Please use the space provided to explain your answers to the questions above. You may also include additional comments for the author, including concerns about dual publication, research ethics, or publication ethics. (Please upload your review as an attachment if it exceeds 20,000 characters)***

***Reviewer #1: (No Response)***

***Reviewer #2: I appreciate the authors for revising the manuscript and making clarifications. I am still not convinced as to why the study seeks to routinize an intervention with unproven efficacy. Is an implementation research on CCP not premature at this time? Given that the above concerns are philosophical rather than methodological, the editor would be in the best position to decide on this.***

We appreciate the reviewer’s question, and we agree that the concern raised has to do with perspective rather than the methodology or interpretation of the reported results. The main issue here has to do with a re-iteration of the intention of our study, which was to evaluate a novel pandemic response program during the height of COVID-19 at an academic medical center in the United States. In response to prior comments raised by Reviewer #2, we have deliberately framed the paper as a program evaluation rather than a formal theory-driven implementation research study of introduction of an established evidence-based practice into a routine healthcare setting. A separate, and somewhat philosophical, question is whether our program evaluation falls under the domain of implementation research. While we are aware that much of implementation research is focused on how best to introduce proven therapies and avail them to target populations, broader definitions of implementation research endorsed by WHO and others are more concerned with the process of implementation itself rather than the evidence-base for the intervention being implemented per se. (For more inclusive broader definitions, see additional references: DH Peters et al., BMJ 2013;347:f6753 doi: 10.1136/bmj.f6753 and Allotey, P., Reidpath, D.D., Ghalib, H. *et al.* Efficacious, effective, and embedded interventions: Implementation research in infectious disease control. *BMC Public Health* 8, 343 (2008). <https://doi.org/10.1186/1471-2458-8-343>; we have added these references to the manuscript - see line 660, references #49 and #50). In the case of CCP, by unprecedented necessity, this therapy was rolled out and widely distributed *before clinical effectiveness* could be established (see lines 97-100). Thus, our objective in this paper is not, in any way, to endorse that CCP is a proven clinically effective therapy, or to tout this work as implementation research, but rather to fill a gap in the literature, that to the best of our ability evaluates the local implementation of emergency use therapies amidst an unprecedented pandemic from a novel rapidly spreading and lethal virus that strained healthcare systems around the globe. Thus, we patterned the evaluation of our program after the dimensions of RE-AIM, consistent with prior applications of this framework and independent from considerations of the *clinical effectiveness* of the therapy (See our reference #15: Glasgow RE, Estabrooks PE. Pragmatic Applications of RE-AIM for Health Care Initiatives in Community and Clinical Settings. Prev Chronic Dis 2018; 15:170271. DOI: [https://doi.org/10.5888/pcd15.170271](https://nam04.safelinks.protection.outlook.com/?url=https%3A%2F%2Fdoi.org%2F10.5888%2Fpcd15.170271&data=05%7C01%7Chroot%40montefiore.org%7Ca8b2096df9ce41e9480508da9a6b2877%7C9c01f0fd65e040c089a82dfd51e62025%7C0%7C0%7C637992082306016851%7CUnknown%7CTWFpbGZsb3d8eyJWIjoiMC4wLjAwMDAiLCJQIjoiV2luMzIiLCJBTiI6Ik1haWwiLCJXVCI6Mn0%3D%7C3000%7C%7C%7C&sdata=Cps8baIVEjSPJnVd4XeyuYXzV4rV%2FecfUObQ9RoOfEE%3D&reserved=0)) (see lines 100-103). While we patterned our program evaluation after RE-AIM to ensure its rigor, we acknowledge that there are few frameworks with flexible construct definitions and a track record of use ideally suited for evaluating pandemic response programs like ours. We use CCP here as a case example to address how to implement emergency therapies for novel and high-mortality pathogens during global pandemics (see discussion lines 517-528).

The FDA endorsement of emergency use CCP resulted in a broad sweeping deployment of CCP to more than 500,000 patients with Covid-19 in the United States alone. While these high numbers establish that CCP can be collected in large volumes and widely distributed throughout the US healthcare system, there is no existing literature that objectively reports what was necessary to implement a CCP program at a local level and whether the ‘expert opinion-based’ recommendations of treatment within certain timeframes of symptoms onset, or at a minimum defined antibody titer level, or via a compassionate use mechanism versus a typically more labor-intensive randomized control trial were feasible for hospitals with surging cases of Covid-19. From seasonal influenza, to Ebola, to now Covid-19, convalescent plasma has surfaced in the emergency setting. Therefore, we think it is critical that an evaluation of a program like ours be performed and reported. As indicated by Reviewer #3, we hope our study will inform future operations that may again turn to convalescent plasma for other novel pathogens, and the lessons learned can help guide expectations and resource allocations if convalescent plasma is used again.

To be very clear, it is not our objective to seek to ‘routinize CCP’ as a clinical intervention. The reviewer makes an important point about the typical order in which the therapeutic pipeline functions—from proven therapy to distribution of that therapy. However, the reality of emergency use therapies for Covid-19 were that CCP was widely distributed *before* clinical effectiveness could be established. We are careful throughout the manuscript therefore to indicate that our report is not meant to be an endorsement of *clinical effectiveness.* Rather, our aim here, again, acknowledging the realities of the pandemic, is to provide as objective of an assessment as possible of the implementation of putative therapies. Evaluating *how* we *implemented* this emergency therapy for a rapidly surging pandemic is critical for informing, and improving upon, future pandemic responses. To that extent, we were uniquely positioned to compare certain metrics between a compassionate use versus a randomized control trial mechanism. These metrics included considerations of the reach of Covid-19 therapeutics and mitigating inequities in access to Covid-19 treatment for historically marginalized populations—both priority areas for the field of implementation research to enhance implementation in the era of Covid-19 (see reference Wensing, M., Sales, A., Armstrong, R. *et al.* Implementation science in times of Covid-19. *Implementation Sci* **15**, 42 (2020). <https://doi.org/10.1186/s13012-020-01006-x>; we have added this reference to the manuscript see line 661, reference #51).

For our present study, we respectfully request that if the editorial staff and other reviewers consider our report appropriate for review and publication in PLoS One, we can continue moving the manuscript forward in a timely manner. We appreciate the debate Reviewer #2’s comments have raised within and external to our group and are open to the potential opportunity for engaging in viewpoint/editorial discussions where these matters of perspective in the field can more fully be put into conversation. What is the role of implementation research in shaping our understanding of *how* to best implement emergency use therapies if the field is restricted only to proven effective therapies? How do governments and policy makers make informed decisions about resource allocations without knowing the personnel and material ‘costs’ of emergency use programs? How do experts provide rational guidance regarding the timing, dosing, and administration of therapies like CCP if there is no understanding of the real-world practicalities of the recommendations? How do panels decide if a situation truly warrants bypassing the randomized control trial mechanism if there has not been an objective comparison of implementation of a randomized control trial compared to compassionate use therapy. A lesson from the Covid-19 response is that decisions to roll-out emergency use therapies can occur at a national level, and prior to knowing if they are clinically proven. How do we best implement these programs when circumstances like an unprecedented pandemic force our healthcare system to operate outside of the typical therapeutic pipeline?

***Further, I maintain that the indicators used for the "effectiveness" component of the Reach-Effectiveness-Adoption-Implementation-Maintenance (RE-AIM) framework do not in anyway speak to "effectiveness". rather they speak more to "Reach". So is the RE-AIM therefore an appropriate framework for this study, given that the intervention has not been proven to be an efficacious treatment, and thus may not be "effective" in the real world.

For clarity here are the measures the authors have for "Effectiveness" the CCP

• The proportion of patients at UNCMC who received CCP within 10 days of symptom onset in the EAP era.
• Proportion of patients who received CCP infusion within 72 hours of admission at UNCMC versus affiliate hospitals in the EAP era.
• Time to remdesivir in CCP recipients and non-recipients at UNCMC in the EAP era.

Clearly these indicators cannot test effectiveness, rather they are mostly measures of "Reach"

Once again, I invite the editor to consider these concerns.***

We have carefully considered this concern. Again, we would like to clarify that when we are referring to “effectiveness” in our manuscript, we are referring to “*implementation effectiveness*”, not “*clinical effectiveness*.” To quote the creator of RE-AIM, Russell Glasgow: “The efficacy-based research paradigm that dominates our current notions of science is limiting and not always the most appropriate standard to apply. A reductionistic scientific paradigm oversimplifies reality in the quest to isolate efficacious treatments. Most clinical trials focus on eliminating potential confounding variables and involve homogeneous, highly motivated individuals without any health conditions other than the one being studied. This approach provides important information and strong internal validity; from an external validity perspective, however, it results in samples of non-representative participants and settings” (Reference: Glasgow et al. Am J of Public Health. September1999, Vol.89, No.9).

In an effort to clarify the definition of implementation effectiveness further, we include here a table from the originator of RE-AIM, Russell Glasgow. This definition of effectiveness is outlined in his table copied and pasted below:

Reference: Glasgow RE, Estabrooks PE. Pragmatic Applications of RE-AIM for Health Care Initiatives in Community and Clinical Settings. Prev Chronic Dis 2018; 15:170271. DOI: [https://doi.org/10.5888/pcd15.170271](https://nam04.safelinks.protection.outlook.com/?url=https%3A%2F%2Fdoi.org%2F10.5888%2Fpcd15.170271&data=05%7C01%7Chroot%40montefiore.org%7Ca8b2096df9ce41e9480508da9a6b2877%7C9c01f0fd65e040c089a82dfd51e62025%7C0%7C0%7C637992082306016851%7CUnknown%7CTWFpbGZsb3d8eyJWIjoiMC4wLjAwMDAiLCJQIjoiV2luMzIiLCJBTiI6Ik1haWwiLCJXVCI6Mn0%3D%7C3000%7C%7C%7C&sdata=Cps8baIVEjSPJnVd4XeyuYXzV4rV%2FecfUObQ9RoOfEE%3D&reserved=0).

For the purposes of implementation effectiveness of our program, the ‘benefit’ we were trying to achieve was timely administration of CCP within the expert-recommended guidance at the time of our program and as measured across sub-groups, like those that did or did not receive other Covid-19 directed therapies. We have referenced this manuscript (see lines 103, 151, 515, 522).

***Reviewer #3: This manuscript is a revised version of a manuscript which describes a retrospective evaluation of a COVID-19 convalescent plasma (CCP) program implemented at a single highly resourced academic medical center. Although this appears to be a relatively small program (including 163 people who received CCP), these data and associated narrative summary of the CCP program may provide considerations for the use of experimental therapeutics during a pandemic. The investigators report metrics describing the success of the framework of their local program and the performance of their local program, particularly relative to affiliate hospitals in a similar catchment area. This is a novel study with important implications for the next global health calamity.

The authors should be commended on the many revisions, which appear to have improved the potential quality and impact of this manuscript— kudos. The manuscript is well-written and nicely organized, and the scientific approach remains high quality. This manuscript offers sage lessons learned and ideas about preparation for future outbreaks of infectious disease.

I have no major criticisms of the work. In the continued spirit of enhancing the quality of the manuscript, modest revisions are suggested below. Please do note the text below represents suggestions and not directives.***

Thank you for your suggestions and for noting that this is a “novel study with important implications for the next global health calamity.” We very much appreciate your additional comments that will enhance the quality of our manuscript. We have responded to each of your suggestions below.  ***Suggested Revisions

Please note the line numbers are based on the revised manuscript with track changes.

Lines 96-98: Should “reduce the demand on large blood donation banks” be changed to “reduce the demand on national blood donation banks”? Of note, there may also be clinical benefits associated with using locally-sourced plasma that may represent regionally-specific, prevalent SARS-CoV-2 variants (PMID: 34381030).***

We have made this edit as well as added the suggested additional comment and reference (see lines 94-96, reference #14). ***Line 222-224: Tittered should likely be replaced with titered. Consider rephrasing this sentence, perhaps, “…to provide sufficient supply of CCP with specified nAb titer”.***

We have made this suggested edit (see line 228). ***Lines 242-248: Consider a comment stating that p-values for adjusted for multiplicity.***

We have added this comment (see line 251). ***Lines 256-259: If a result is not statistically significant, there is limited empirical evidence to support the statement. Consider removing this sentence.***

We have removed this part of the sentence (see lines 259-260). ***Table 2. As noted in the discussion, fantastic inclusion of members of minority race and ethnic groups. Interesting to see reduced representation of minority groups in the RCT relative to the overall program.***

Thank you for your comment. We agree that this is an interesting observation.  ***Lines 275-276: Consider adding the phrase “of symptom onset” to the end of the sentence, such that the sentence may read “…were treated within 10 days of symptom onset”.***

We have added this phrase to the end of that sentence (See line 278). ***Lines 281-284: Consider adding “of admission” after 48 hours and 24 hours.***

We have done this (see line 288). ***Lines 281-286: Consider switching the order of sentences 2 and 3, such that the sentence that begins “The median time from…” precedes the sentence beginning “Comparing patients receiving…”***

We have switched the order of these two sentences (see lines 283-288). ***Lines 284-286: The second use of ‘median’ appears to be redundant. Consider rephrasing.***

We have rephrased this sentence (see line 283). ***Lines 322-326: This is an important and interesting finding. Although ABO compatible plasma should be transfused preferentially. Did the group use either Group A plasma or low anti-A titer Group O plasma if ABO-compatible plasma was not available?***

We are aware that exceptions were allowed for use of these low anti-blood group antigen titer plasma if ABO-compatible plasma was unavailable. Out of abundance of caution and for safety purposes, however, we designed our program to only infuse ABO-compatible plasma.  ***Lines 329-330: The additional time burden was associated with CCP, correct? Although this may be intuited, consider clearly denoting.***

We have edited this sentence to more clearly denote that (see lines 336-337). ***Line 392: Although 8.5 donors may be mathematically precise, referring to a portion of (rather than a whole) person is “odd”.***

We have changed this to “…approximately 8 donors…” (see line 403).
